# Discovery and characterization of stable and toxic Tau/phospholipid oligomeric complexes

**DOI:** 10.1038/s41467-017-01575-4

**Published:** 2017-11-22

**Authors:** Nadine Ait-Bouziad, Guohua Lv, Anne-Laure Mahul-Mellier, Shifeng Xiao, Gizem Zorludemir, David Eliezer, Thomas Walz, Hilal A. Lashuel

**Affiliations:** 1 Laboratory of Molecular and Chemical Biology of Neurodegeneration, Station 19, Ecole Polytechnique Fédérale de Lausanne, CH-1015 Lausanne, Switzerland; 2 Department of Biochemistry and Program in Structural Biology, Weill Cornell Medicine, 1300 York Avenue, New York, NY 10021 USA; 3 Laboratory of Molecular Electron Microscopy, Rockefeller University, 1230 York Avenue, New York, NY 10065 USA; 4 Present Address: Shenzhen Key Laboratory of Marine Biotechnology and Ecology, College of Life Sciences and Oceanography, Shenzhen University, 518060 Shenzhen, China

## Abstract

The microtubule-associated protein Tau plays a central role in the pathogenesis of Alzheimer’s disease. Although Tau interaction with membranes is thought to affect some of its physiological functions and its aggregation properties, the sequence determinants and the structural and functional consequences of such interactions remain poorly understood. Here, we report that the interaction of Tau with vesicles results in the formation of highly stable protein/phospholipid complexes. These complexes are toxic to primary hippocampal cultures and are detected by MC-1, an antibody recognizing pathological Tau conformations. The core of these complexes is comprised of the PHF6* and PHF6 hexapeptide motifs, the latter in a β-strand conformation. Studies using Tau-derived peptides enabled the design of mutants that disrupt Tau interactions with phospholipids without interfering with its ability to form fibrils, thus providing powerful tools for uncoupling these processes and investigating the role of membrane interactions in regulating Tau function, aggregation and toxicity.

## Abstract (web summary)

The Alzheimer protein Tau interacts with biological membranes, but the role of these interactions in regulating Tau function in health and disease remains unexplored. Here, the authors report on the discovery and characterization of neurotoxic oligomeric protein/phospholipid complexes.

## Introduction

**Fig. 1:**
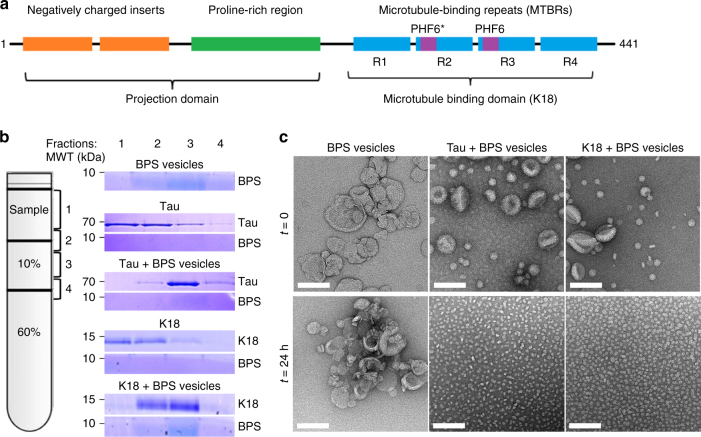
Tau and K18 bind to and disrupt vesicles formed with negatively charged phospholipids, resulting in the formation of globular Tau/K18-phospholipid complexes. **a** Schematic depiction of the Tau sequence. The longest Tau isoform consists of an N-terminal projection domain containing two negatively charged inserts, N1 and N2 (orange), and a proline-rich region (green), and a C-terminal microtubule-binding domain with four microtubule-binding repeats, R1−R4 (blue). R2 and R3 contain the hexapeptides PHF6* and PHF6 (purple), respectively, which are necessary to drive Tau fibrilization. **b** Left: schematic representation of the vesicle sedimentation assay set-up; fraction 1: sample containing no sucrose, fraction 2: interface between sample and 10% sucrose solution, fraction 3: 10% sucrose solution, fraction 4: interface between 10 and 60% sucrose solutions. Right: SDS-PAGE analysis of the different fractions for different samples. **c** Negative-stain EM images of BPS vesicles alone (left) and mixed with Tau (middle) and K18 (right) at a molar protein:phospholipid ratio of 1:20 immediately after mixing (top panels) and after 24 h of incubation (bottom panels). The scale bars are 100 nm

In the brain of patients suffering from Alzheimer’s disease (AD) and other neurodegenerative diseases^1,2^, the microtubule-associated protein Tau is found in various aggregated forms, including soluble oligomers and highly insoluble β-sheet-rich fibrils, the paired helical filaments (PHFs)^3^. Although the exact sequence and molecular determinants of Tau aggregation remain unknown, two sequence characteristics of the protein have been proposed to play key roles in this process: (1) the highly positive charge of its basic C terminus, which requires compensation by polyanions^4,5^, and (2) its intrinsic propensity to form a β-structure^6^, which is thought to be driven by two hydrophobic hexapeptides, PHF6 (VQIVYK) and PHF6* (VQIINK), in the second and third of the four microtubule-binding repeats, R1−R4 (Fig. 1a). Several lines of evidence also suggest that interactions of Tau with membranes may affect some of its physiological functions and its aggregation properties. Under physiological conditions, Tau is known to bind to the plasma membrane of neuronal and non-neuronal cells in a phosphorylation-dependent manner and through specific interactions involving both the N-terminal region and the microtubule-binding domain^7–14^. The association of Tau filaments with plasma membranes in AD brains provided the first indication that membranes could provide an environment that promotes Tau oligomerization and subsequent assembly into PHFs^15^. Subsequent in vitro studies demonstrated that interactions of Tau with membranes, anionic vesicles and micelles facilitate its aggregation and modulate different aspects of its fibril formation pathway^16–21^. Conversely, the process of Tau fibrillization on membranes has been shown to lead to disruption of membrane dynamics and/or integrity through different mechanisms. These observations are consistent with previous studies demonstrating the presence of various phospholipids in brain-derived Tau filaments and support a pathological role of Tau-membrane interactions in PHF formation and AD^22^.

Here, we investigate the sequence determinants and consequences of the interactions of the longest Tau isoform (2N4R), the K18 fragment, a C-terminal Tau fragment containing its four microtubule-binding repeats, and other Tau isoforms with membranes with the aims (1) to assess the potential effects of Tau on membranes, (2) to characterize the biophysical, cellular and toxic properties of the macromolecular species arising from these interactions, and (3) to identify mutants that disrupt Tau interactions with membranes without interfering with its ability to form fibrils. Such mutants could serve as powerful tools to uncouple these two processes and to investigate the effect of membrane interactions on the function, aggregation and toxicity of Tau in primary neuron cultures.

## Results

### Tau and K18 interact with negatively charged vesicles

To investigate whether Tau and K18 could interact with acidic vesicles composed of porcine brain phosphatidylserine (BPS), we first used a vesicle sedimentation assay (Fig. 1b). In this assay, unilamellar vesicles were formed, mixed with the proteins and then applied to a sucrose step gradient. After centrifugation, soluble proteins remain on top of the gradient, whereas vesicles migrate into the gradient. The distribution of the proteins and vesicles, as well as vesicle morphology, were then evaluated by SDS-PAGE (Fig. 1b) and negative-stain electron microscopy (EM) (Supplementary Fig. 1). When run individually, BPS vesicles migrated in the lower fractions of the gradient, whereas, as expected for soluble proteins, Tau and K18 did not exhibit significant migration. However, when mixed with BPS vesicles, both Tau and K18 migrated to lower fractions, indicating that they bound to the vesicles. Tau migrated farther than K18, suggesting that a larger proportion of Tau than of K18 molecules bound to the vesicles, or that Tau bound more tightly to the vesicles, possibly through the projection domain, which is known to interact with membranes and is missing in K18^7–12^.

### Tau forms stable protein/phospholipid complexes

**Fig. 2:**
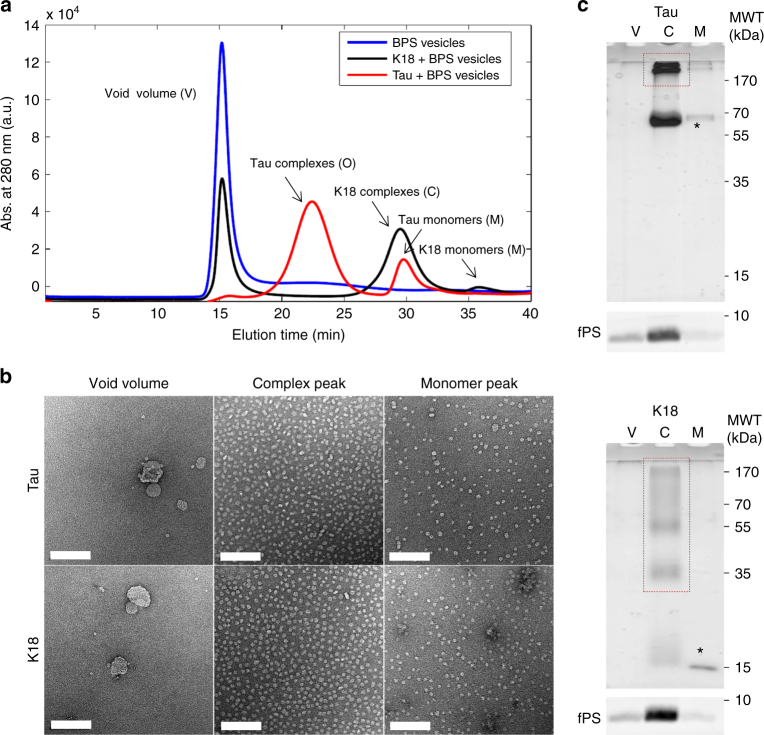
Characterization of the Tau and K18 protein/phospholipid complexes. **a** Chromatograms of BPS vesicles alone (blue) and after incubation for 24 h with K18 (black) and Tau (red) at a molar protein:phospholipid ratio of 1:20. The samples were run on a Superose 6 column at a flow rate of 0.5 ml min^−1^. Vesicles eluted in the void volume (15 min), globular particles formed by Tau and K18 eluted between 20 and 25 min and between 27 and 32 min, respectively, and monomeric Tau and K18 eluted at 30 min and 36 min, respectively. **b** Negative-stain EM images of the void volume, the globular particles, and monomer fractions of Tau (top) and K18 (bottom). The scale bars are 100 nm. **c** SDS-PAGE gel of the void volume (V) and the peaks representing the protein/phospholipid complexes (C) and the monomers (M) obtained with Tau (top) and K18 (bottom). The asterisks mark bands of monomeric proteins and the dashed red rectangles indicate oligomeric species. The phospholipids, containing 1% NBD-labeled phospholipids, which were imaged separately with a Typhoon trio fluorescent scanner, are shown on the bottom (labeled fPS)

**Fig. 3:**
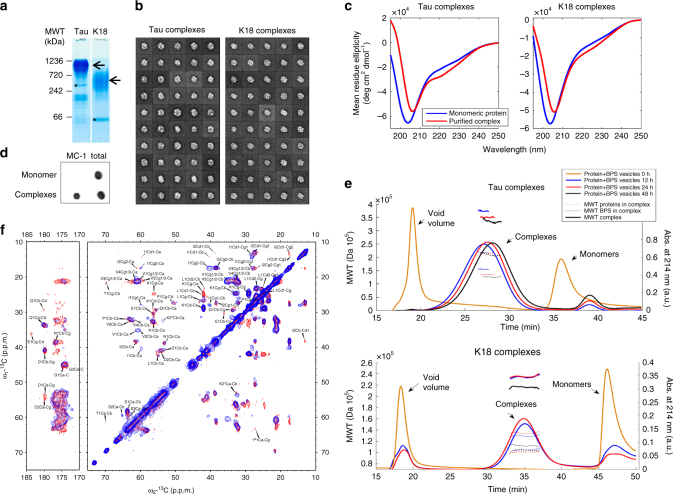
Structural characterization of Tau and K18 protein/phospholipid complexes. **a** NativePAGE analysis of protein/phospholipid complexes formed by Tau (left) and K18 (right) after 24 h of incubation with BPS vesicles at a molar protein:phospholipid ratio of 1:20. The monomer bands are indicated by asterisks, and the presence of higher molecular weight bands, indicated by arrows, demonstrates that the protein/phospholipid complexes contain oligomerized proteins. **b** Averages obtained from the classification of ~4850 particles of Tau with a radius ranging from 100 to 110 Å and ~4000 particles of K18 with a radius ranging from 81 to 97 Å into 50 classes. **c** CD spectra of Tau (left) and K18 (right) alone (blue) and their phospholipid complexes (red) formed by incubation with BPS vesicles at a molar protein:phospholipid ratio of 1:20. The formation of protein/phospholipid complexes is accompanied by a slight increase in secondary structure content. **d** Dot blots (0.25 µg) of Tau monomer or Tau incubated for 4 days with BPS vesicles at 37 °C. Tau was detected using the MC-1 antibody (dilution 1:500), which recognizes pathological Tau conformations, and a homemade rabbit polyclonal anti-Tau antibody (dilution 1:5000) to assess the total amount of protein. **e** Determination of the molecular weight of protein/phospholipid complexes formed by Tau and K18 by multi-angle light scattering. Although the number of subunits in the complex remains constant (5 for Tau and 7–8 for K18), the number of phospholipid decreases over time (from ~190 to ~150 for Tau and from ~160 to ~140 for K18). The final molecular weight of the protein/phospholipid complexes formed by Tau and K18 are ~335 kDa and 230 kDa, respectively. **f** Residue type assignments of [U-13C/15N]-labeled K18/phospholipid complexes using 2D DARR solid-state NMR spectra. Mixing times for DARR are 20 ms (blue) and 150 ms (red). Both spectra were recorded at 17.6 T, 278 K, and 13 kHz magic-angle spinning

We next assessed the effect of both proteins on vesicle morphology as a function of time. We observed that co-incubation of Tau and BPS vesicles at a molar protein:phospholipid ratio of 1:20 resulted in the generation of homogenous globular structures. EM analysis showed that BPS vesicles are typically heterogeneous in size and have a tendency to clump together (Fig. 1c). The addition of Tau or K18 induced immediate changes in the vesicles, which separated and became more homogeneously small and round. Over the course of a few hours, the vesicles almost completely disappeared and were replaced by globular particles of homogeneous size. The appearance of the globular particles correlated with the disappearance of the BPS vesicles, suggesting that they may contain phospholipids. To test this hypothesis, we incubated Tau and K18 with BPS vesicles containing NBD-labeled fluorescent phospholipids, isolated the resulting Tau/phospholipid complexes by size-exclusion chromatography (SEC), and probed their composition by SDS-PAGE (Fig. 2). Fluorescence scanning of the SDS-PAGE gels revealed that the globular particles contained both proteins and phospholipids. These particles were stable over a wide range of ionic strength and even at high temperatures (Supplementary Fig. 2a−c), and on native gels the particles ran as high-molecular-weight species (Fig. 3a, arrows). Similar structures were detected at Tau concentrations of 100–500 nM (Supplementary Fig. 2d), which are below the physiological concentration of Tau in neurons (1–2 μM)^23,24^.

To gain further insights into the structure and size distribution of the Tau and K18/phospholipid complexes, we imaged the particles by negative-stain EM and performed single-particle analysis. Although the resulting class averages show that the Tau and K18 particles are quite homogenous in size, they do not appear to have defined structural features (Fig. 3b). The circular dichroism (CD) spectra of the Tau and K18 protein/phospholipid complexes revealed a slight increase in secondary structure contents, compared to those of the proteins alone (Fig. 3c).

To determine whether the Tau/phospholipid complexes share structural characteristics with pathological Tau aggregates, we assessed their immunoreactivity to antibodies that are specific for pathological forms of Tau. MC-1 is a monoclonal antibody raised against Tau PHFs that were purified from AD brain homogenates^25^. Notably, MC-1 recognized purified Tau/phospholipid complexes (Fig. 3d), suggesting that Tau in these phospholipid complexes could bear structural characteristics that occur in Tau during the development of AD pathology.

To further assess the size distribution and composition of the protein/phospholipid complexes, Tau and K18 were incubated with BPS vesicles and the samples were analyzed by multi-angle light scattering (MALS) after different time periods (Fig. 3e). The total mass, the mass of the protein and the mass of the phospholipids in the complexes were followed over time using the Astra 5.3 analysis software^26^. We observed that the complexes tended to release a small amount of phospholipids over time, whereas the number of proteins per complex remained essentially constant. After reaching equilibrium, the average protein:phospholipid ratios were calculated to be 5:150 for Tau/phospholipid complexes (molecular weight = 335 kDa) and 7−8:140 for K18/phospholipid complexes (molecular weight = 230 kDa).

Next, we investigated whether Tau and K18 protein/phospholipid complexes could form with vesicles that more closely resemble the lipid composition of native neuronal membranes. Mixing Tau or K18 with vesicles formed with total porcine brain lipid extract (TBE), which contains ~10% of phosphatidylserine, resulted in the formation of Tau and K18 protein/phospholipid complexes, albeit with lower efficiency (Supplementary Fig. 3a, top panels). However, increasing the ratio of phospholipids to protein to 160:1 (mol:mol) significantly enhanced Tau binding to the TBE vesicles and promoted the formation of Tau and K18 protein/phospholipid complexes as determined by CD, sedimentation assay and EM (Supplementary Fig. 3), indicating that Tau binds to the negatively charged phospholipids present in the TBE membrane. Taken together, these data suggest that, under near-physiological conditions, Tau is able to bind to negatively charged lipids in mixed membranes and to extract them to form stable Tau/phospholipid complexes.

**Fig. 4:**
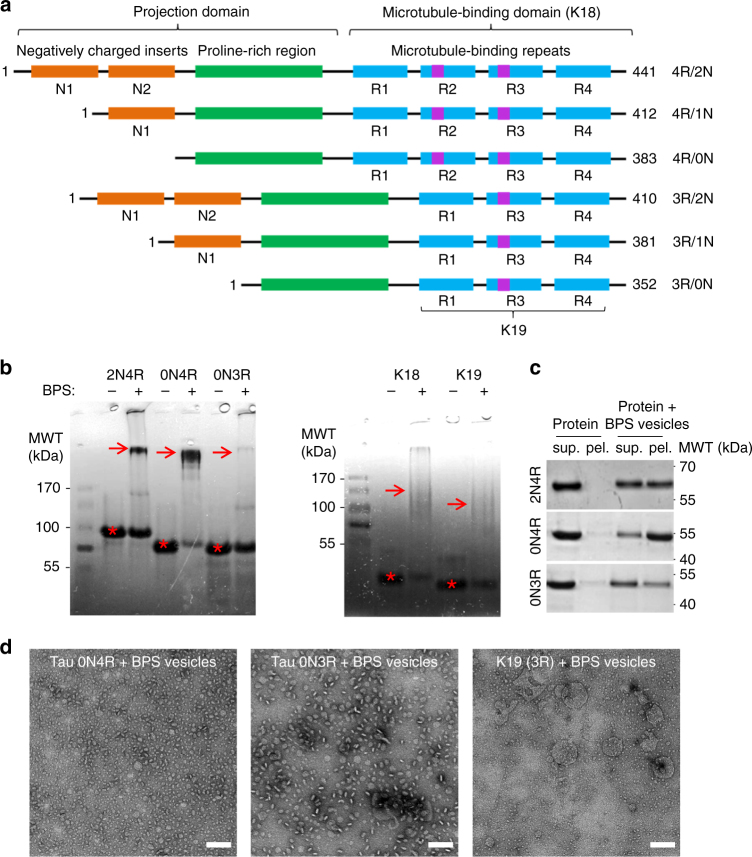
Tau isoforms lacking the R2 domain are deficient in the formation of protein/phospholipid complexes. **a** Human Tau naturally occurs in six isoforms, depending on the alternative splicing of exons E2, E3, and E10, giving rise to the variants containing either 0, 1, or 2 N-terminal inserts (0N, 1N, and 2N), and 3 or 4 microtubule-binding repeats (3R and 4 R). **b** NativePAGE analysis of protein incubated alone or with BPS vesicles at a molar protein:phospholipid ratio of 1:20. The Tau 0N4R isoform was able to form protein/phospholipid complexes as efficiently as the WT protein, while Tau 0N3R and K19 formed significantly less complexes. The monomer bands, are indicated by asterisks, and the presence of higher molecular weight bands are indicated by arrows. **c** Co-sedimentation assay of BPS vesicles mixed with the three Tau isoforms (from top to bottom: Tau 2N4R, Tau 0N4R, and Tau 0N3R) at a molar protein:phospholipid ratio of 1:100. When incubated alone (left), all proteins remained in the supernatant (sup.), but in the presence of vesicles (right), the proteins partially co-sedimented with the vesicles to the pellet fraction (pel.), indicative of membrane binding. Tau 2N4R and Tau 0N4R co-sedimented more than Tau 0N3R. **d** EM analysis of Tau 0N4R (left panel), Tau 0N3R (middle panel) and K19 (right panel) mixed with BPS vesicles at a molar protein:phospholipid ratio of 1:20 showed that Tau 0N4R can form protein/phospholipid complexes, while Tau 0N3R formed some large round and bean-like structures, and K19 formed few complexes with many vesicles remaining. The scale bars are 100 nm

To determine whether other natural Tau isoforms (Fig. 4a) exhibit different propensity to interact with membranes, we investigated the ability of recombinant K19 (3R), Tau 0N4R, and Tau 0N3R to interact with BPS vesicles and to form complexes with phospholipids under the same conditions used for Tau and K18. We observed that Tau 0N4R formed complexes with phospholipids, as evidenced by the oligomeric band apparent on native gels (Fig. 4b, arrow), the significant binding of Tau 0N4R to BPS vesicles in the co-sedimentation assay (Fig. 4c), and the observation of globular particles in EM images (Fig. 4d). Interestingly, Tau 0N3R, which lacks the R2 repeat domain, exhibited modest interactions with vesicles, did not cause complete vesicle disruption, and was less efficient in forming complexes with phospholipids. On the other hand, K19, which also lacks the R2 repeat, seems to retain the ability to form protein/phospholipid complexes, albeit to a lesser extent than Tau 2N4R and K18. These results imply that repeat R2 plays an important role in the formation and stabilization of Tau/phospholipid complexes, while the N-terminal inserts do not appear to be essential for the formation of Tau/phospholipid complexes, as already suggested by the high propensity of K18 to disrupt BPS vesicles and form complexes with phospholipids.

### PHF6*/PHF6 form the core of the protein/lipid complexes

**Fig. 5:**
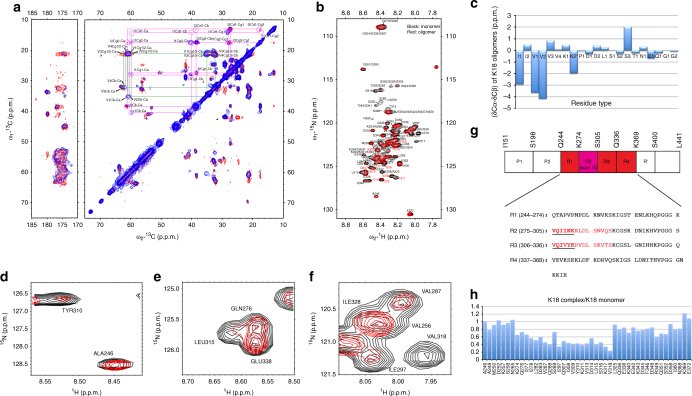
Identification of residues that form the K18/phospholipid oligomer core. **a** Valine and isoleucine spin system assignments showing the presence of two Val residues and one Ile residue in a β-strand conformation (signals shifted down and right reflect decreased Cα and increased Cβ shifts characteristic of β-strand). **b** Proton-nitrogen HSQC spectrum of a K18/phospholipid complex preparation in solution (red), compared with a matching spectrum of the monomeric protein (black). Resonance assignments are indicated, with red labels for the PHF6 and PHF6* regions. **c** Secondary carbon chemical shifts (Cα-Cα_rc + Cβ-Cβ_rc) by amino acid type for signals observed in DARR spectra of K18/phospholipid complexes. Lys-2 is marked with an asterisk to indicate that this tentatively assigned lysine residue exhibits chemical shifts consistent with a pre-proline residue, indicating that it corresponds to residue Lys-311. **d**–**f** Regions from the proton-nitrogen correlation HSQC spectra of complexes suspended in solution (red), compared with matching spectra of the monomeric protein (black). The signals from the PHF6 region (Tyr-310 and Leu-315), from the PHF6* region (Gln-276), and from the ~9 residues subsequent to each PHF motif (Val-287 and Val-318) are highly attenuated, indicating their immobilization in the oligomer core. Signals from other regions of K18 are much less affected, indicating that they remain highly flexible outside the oligomer core. Signals from Ile-297 and Ile-328 also show some attenuation, possibly resulting from their close proximity to regions within the core, which likely restricts their mobility. **g** Schematic of the K18 sequence and its location within full-length Tau, highlighting in red the residues that appear to form the oligomer core. The hexapeptide motifs PHF6 and PHF6* are underlined and in bold type. **h** Ratios of intensities for well resolved signals in HSQC spectra of protein/phospholipid complexes versus monomers, showing that the PHF6 region and subsequent ~9 residues, are most highly attenuated, but that the corresponding region in repeat 2, including the PHF6* motif and subsequent ~9 residues are also significantly attenuated compared to the remainder of the protein

To probe the conformation of Tau in the protein/phospholipid complexes, we applied solid-state and solution Nuclear Magnetic Resonance (NMR) to 13C/15N-enriched K18/phospholipid complexes. Carbon-carbon correlation (DARR) spectra at short and long NMR mixing times (Figs. 3f and 5a) exhibit signals arising from well-ordered rigid protein regions and allow identification of their amino acid types, even in the absence of sequence-specific assignments. In contrast, proton-nitrogen correlation (HSQC) spectra exhibit signals from highly flexible regions (Fig. 5b). Based on carbon chemical shifts (Fig. 5c), four well-ordered residues exhibited a marked secondary structure preference, one isoleucine, one lysine and two valines. Because secondary structure by definition must be contiguous over several residues, and the only location in K18 where these four residues occur in close proximity is the PHF6 motif (VQIVYK), we assigned these residues to Val-306, Ile-308, Val-309, and Lys-311, indicating that within the oligomer core, the PHF6 motif adopts an extended β-strand-like secondary structure. Furthermore, the signals assigned to residue Lys-311 exhibit carbon chemical shifts that are uniquely associated with a location just before a proline residue, and the only Lys–Pro dipeptide in K18 is Lys-311–Pro-312, strongly supporting our assignment. Well-resolved resonances from the PHF6 motif are also strongly attenuated in the HSQC spectra (Fig. 5d, e, h), confirming their immobilization in the oligomer core. This assignment is consistent with the PHF6 motif being the most critical for nucleating Tau filaments and having the strongest predisposition to adopt β-strand conformation^27^.

The amino acid identities of the remaining DARR signals suggest that the oligomer core also includes the PHF6* motif, as well as at least 9 residues following each hexapeptide motif. Inclusion of PHF6* is justified by at least one additional isoleucine signal, likely from Ile-277 and/or Ile-278, and at least one asparagine residue, likely Asn-279. Importantly, resonances from PHF6* are also somewhat attenuated in the HSQC spectrum (Fig. 5e, h). It should be noted, however, that this pair of residues is also found further along the R2 repeat, Asn-296 and Ile-297, and their signals are also attenuated in the HSQC spectrum (Fig. 5f, h). Hence, inclusion of PHF6* in the oligomer core, while reasonable, is less certain than that of PHF6. Additional assignments include a threonine residue, likely Thr-319, at least 2, probably 3–4 additional valine signals, likely Val-275, Val-313, Val-287 and/or Val-318, and at least three serine signals, likely Ser-285, Ser-316, Ser-289, and/or Ser-320. Residues Val-287 and Val-318 are significantly attenuated in the HSQC spectrum (Fig. 5f, h), supporting these assignments. Two glycine signals suggest that the KCGS motifs (residues 290–293 and 321–324) might be part of the oligomer core as well.

### Tau/phospholipid complexes promote Tau internalization

**Fig. 6:**
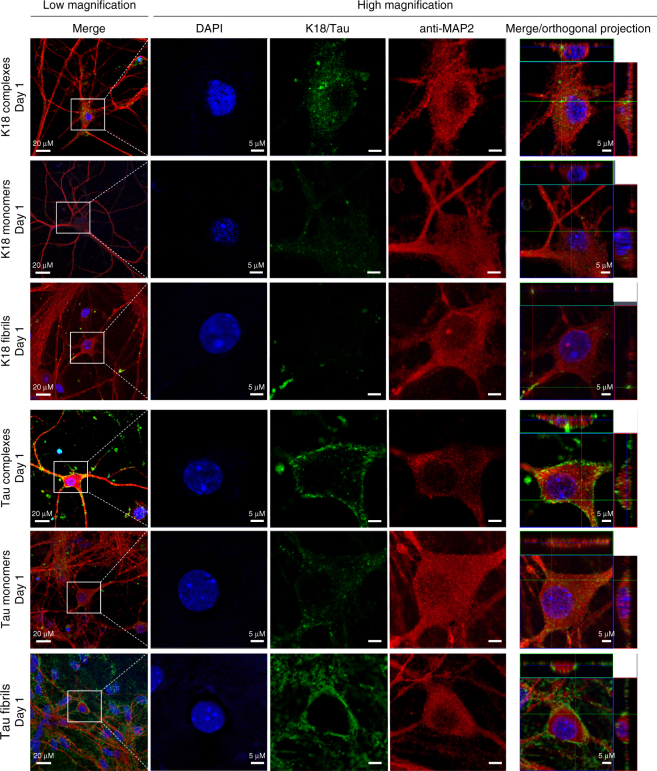
Neuronal internalization of Tau and K18 monomers, fibrils and protein/phospholipid complexes. Hippocampal primary neurons were treated with PBS, 1 µM Tau or Oregon Green-labeled K18 (K18-OG) protein/phospholipid complexes for 1 day and processed for immunocytochemistry. From left to right: overview image, DAPI staining to show the nucleus (blue), K18-OG or staining with anti-Tau antibody Tau13 (green), staining with anti-MAP2 antibody identifying neurons (red), and orthogonal projections. In neurons treated with protein/phospholipid complexes containing K18-OG, K18 localizes to the plasma membrane and is internalized by primary neurons after 1 day of treatment, as evidenced by the co-staining with MAP2. In neurons treated with monomeric K18-OG, K18 is internalized and diffuse in the cytoplasmic compartment and in small punctae after 1 day of treatment. In neurons treated with K18-OG fibrils, K18 localizes to the membrane in the form of large punctae that are internalized by primary neurons after 1 day of treatment. In neurons treated with Tau/phospholipid complexes, Tau localizes to the plasma membrane and is internalized after 1 day of treatment. In neurons treated with monomeric Tau, Tau is internalized and present diffuse in the cytoplasmic compartment and in small punctae after 1 day of treatment. In neurons treated with sonicated Tau fibrils, Tau localizes to the plasma membrane and is not internalized at 1 day post-treatment. The scale bars are 20 µm (low magnification) and 5 µm (high magnification)

To investigate the interaction of Tau/phospholipid complexes with biological membranes and to compare their internalization into primary neurons with that of monomeric and fibrillar forms of Tau and K18, we treated primary hippocampal mouse neurons with complexes formed by Tau or Oregon Green-labeled K18 with BPS and assessed their uptake by immunocytochemistry (ICC) (Fig. 6, Supplementary Fig. 4). Initially, the fluorescence signal strongly localized to the plasma membrane, but after 1 day of treatment was also seen inside the cell. After 3 days, the cytoplasm was slightly depleted of K18 or devoid of Tau, which was still strongly present at the plasma membrane. These results suggest that the Tau and K18 protein/phospholipid complexes are rapidly taken up by neurons (within 1 day), where they relocalize over time or, for Tau, are possibly degraded. Tau fibrils were not internalized, but deposited at the plasma membrane, while K18 fibrils were readily internalized and observed predominantly as large puncta inside neurons and at the membrane. Unlike the fibrils, the Tau and K18 monomers did not localize to the plasma membrane, exhibited mostly diffuse staining, and appeared occasionally as small punctae in the cytoplasm, albeit to a much lower extent than seen for the corresponding protein/phospholipid complexes. Taken together, these data suggest that the Tau and K18 protein/phospholipid complexes are easily taken up by neuronal cells and may provide a mechanism for transporting Tau between different neurons or neurons and neighboring cells.

To probe for the entry mechanism of the Tau and K18 protein/phospholipid complexes, we performed co-staining with two endolysosomal markers; we used anti-EEA1 and anti-LAMP1 antibodies as markers for early endosomes and late endosomes/lysosomes, respectively. As shown in Supplementary Fig. 5, we observed co-localization of both Tau and K18 protein/phospholipid complexes with EEA1, but not LAMP1. Both Tau and K18 monomers also co-localized with EEA1, but to a lower extent as compared to the Tau and K18 protein/phospholipid complexes. Tau and K18 fibrils did not co-localize with either EEA1 or LAMP1 (Supplementary Figs. 6, 7). These results strongly support the notion that the Tau and K18 protein/phospholipid complexes are taken up through the endocytic pathway, but appear to escape from it, as they are not found in late endosomes or lysosomes.

### Tau complexes can convert into elongated structures

Next, we sought to determine if the Tau/phospholipid complexes represent a stable and potentially functional form of the protein that, under stress conditions or upon changes in the microenvironment, could convert into fibrils. We observed that at slightly acidic pH (6–6.5), the protein/phospholipid complexes readily converted into long tubular and filamentous-like aggregates that exhibit more ordered secondary structure (Supplementary Fig. 8). Moreover, the presence of phospholipids in the pellet fractions following centrifugation suggests that these structures contain Tau bound to phospholipids. These results suggest that changes in pH may be sufficient to initiate Tau polymerization, but the transition to β-sheet-rich fibrils may require additional secondary structure changes that, while not accessible under these conditions, could occur in the cellular environment.

### R2 and R3 and PHF6 mediate interaction of Tau with lipids

To further identify the specific sequence elements of Tau that mediate membrane interaction and the formation of protein/phospholipid complexes, we focused on peptides corresponding to the four microtubule-binding repeats, R1−R4, which have been shown to interact with detergent micelles and vesicles by forming short amphipathic α-helices at the surface of the micelle or membrane^16,17,20^, or in trifluoroethanol^28,29^ (Supplementary Fig. 9a, b).

When incubated alone, R2 and R3 formed β-sheet-rich fibrils, whereas R1 and R4 remained disordered and soluble (Supplementary Fig. 9c, d), consistent with previous reports^29^. Although all microtubule-binding repeats change structure upon binding to membranes, only R2 and R3 formed ThT-positive peptide/phospholipid fibrils (Supplementary Figs. 9c, d and 10), likely due to the presence of the two hexapeptides PHF6* and PHF6 in R2 and R3, respectively. Consistent with this hypothesis, upon incubation with vesicles, the PHF6 peptide formed β-sheet-rich, long and thick fibrils that contained phospholipids (Supplementary Figs. 9c, 10a, b). A more detailed analysis of the interaction of the repeat peptides and PHF6 with membranes can be found in Supplementary Figs. 9, 10. Taken together, these results suggest that upon interaction of Tau with a membrane, one or several of the repeat peptides could form transient α-helices that increase the effective concentration of the PHF6* and PHF6 peptides close to the membrane, which, in turn, could promote Tau/phospholipid complex formation. The helical wheel representations of the repeat peptides R1−R4 reveal the presence of positively charged lysine residues at the interface between the hydrophobic and hydrophilic sides (Supplementary Fig. 9b). These residues could explain why the presence of negatively charged phospholipids is important for the binding of Tau to membranes and the formation of protein/phospholipid complexes.

### Mutations in R2, R3, and PHF6 reduce Tau-membrane binding

**Fig. 7:**
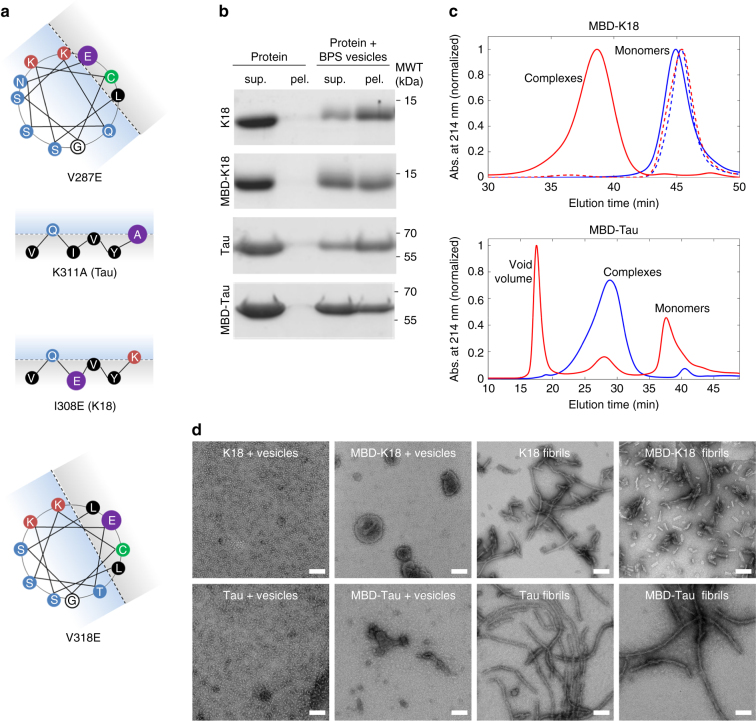
Design and characterization of the membrane binding-deficient K18 and Tau mutants. **a** Helical wheel and β-strands depictions to illustrate the rational for the design of the K18 and Tau mutants. Mutations are in purple, hydrophilic residues in blue, hydrophobic residues in black, positively charged residues in red, cysteine in green, and glycine in white. The hydrophobic and hydrophilic faces of the helices are depicted in light gray and blue, respectively, and the putative position of the negatively charged phospholipid head groups are indicated by dashed lines. Mutation of a hydrophobic Val residue in the hydrophobic part of an amphipathic motif to a negatively charged Glu should interfere with the capacity of the segment to embed itself into the hydrophobic core of a membrane. In the case of Tau, the modification of positively charged Lys-311 to an uncharged Ala should affect its capacity to interact with negatively charged membranes through electrostatic interactions. **b** Co-sedimentation assay of BPS vesicles with WT and MBD-K18 (top) and Tau (bottom). When incubated alone (left), all proteins remain in the supernatant (sup.), but in the presence of BPS vesicles (right), the proteins partially co-sediment with the vesicles to the pellet (pel.), indicative of membrane binding. Compared with the WT proteins, more MBD-K18 and MBD-Tau proteins remain in the supernatant, indicating that they partially lost the ability to bind membranes. **c** Top: size-exclusion chromatograms of WT K18 (solid lines) and MBD-K18 (dashed lines) alone (blue lines) or with BPS vesicles (red lines) show that only WT K18 forms protein/phospholipid complexes. Bottom: size-exclusion chromatograms of WT Tau (blue line) and MBD-Tau (red line) show that WT Tau is efficient in forming protein/phospholipid complexes (blue, large peak) while the capacity of MBD-Tau to form such complexes is compromised (red, small peak). **d** EM analysis of WT and MBD-K18 (top) and Tau (bottom) shows that the mutants cannot form protein/phospholipid complexes in the presence of BPS vesicles (second-to-left panels) but retain their ability to form fibrils (right-most panels). The scale bars are 100 nm

Given our observation that only the R2, R3, and PHF6 peptides converted vesicles into filaments, knowing that they can form amphipathic motifs^29^, and that they are part of the oligomeric core, we explored the possibility of generating mutations that would disrupt membrane binding but not fibril formation. After screening several mutations, we succeeded in generating a K18 mutant, hereafter referred to as membrane binding-deficient K18 (MBD-K18), in which the following hydrophobic residues were mutated to negatively charged glutamate residues: Val-287 and Val-318 in the hydrophobic region of the R2 and R3 amphipathic helix-forming motifs (whose signals were significantly attenuated in the HSQC spectrum), as well as Ile-308 in the hydrophobic region of PHF6 (Fig. 7a and Supplementary Fig. 11a). These mutations did not change the unfolded nature of K18 (Supplementary Fig. 11b). However, unlike for wild-type (WT) K18, incubation of MBD-K18 with BPS vesicles had little to no effect on its structure, indicating a loss of membrane binding (Supplementary Fig. 11b). Membrane binding was also assessed using a sedimentation assay, which showed that less MBD-K18 co-sedimented with the vesicles compared with WT K18 (Fig. 7b). This result showed that the membrane-binding capability of MBD-K18 was significantly reduced but not fully abrogated, possibly due to the remaining amphipathic segments of R1 and R4. While these segments did not convert into filaments in our vesicle incubation assays, they could still mediate some degree of membrane association^16,17,20^. However, the formation of high-MW protein/phospholipid complexes was fully abrogated, as demonstrated by the complete absence of a protein/phospholipid peak in a gel filtration run (Fig. 7c). Negative-stain EM also showed the absence of MBD-K18/phospholipid complexes (Fig. 7d), further demonstrating that only R2, R3, and the PHF6 motif participate in the formation of protein/phospholipid complexes.

To preserve the aggregation behavior of the full-length MBD-Tau mutant, which has a lower propensity to aggregate than K18, the I308E mutation in the PHF6 was replaced with the K311A mutation, as we observed that Tau bearing the I308E does not aggregate. Mutation of Lys-311 to alanine has been reported to have little effect on Tau polymerization in the presence of polyanions^30^, and changing a positively charged lysine residue to an uncharged alanine residue is expected to weaken the interaction of Tau with negatively charged membranes. The capacity of MBD-Tau (V287E, V318E, and K311A) (see Fig. 7a, Supplementary Fig. 11a) to fibrilize in the presence of heparin was not affected (Fig. 7d), whereas its capacity to form protein/phospholipid complexes in the presence of vesicles was strongly inhibited, although not fully abrogated as SEC studies revealed a small but significant peak representing protein/phospholipid complexes (Fig. 7c). EM analysis of the corresponding fractions did not reveal the expected protein/phospholipid complexes but rather showed the presence of small vesicles. Sedimentation assays revealed a significant decrease in the amount of protein that co-sedimented with the vesicles for the mutant compared to the WT protein (Fig. 7b), demonstrating the reduced membrane-binding capacity of MBD-Tau. Incubation of MBD-Tau with BPS vesicles had little effect on its secondary structure, further indicating a loss of membrane binding (Supplementary Fig. 11b). Taken together, these results suggest that sequences outside the microtubule-binding domain of Tau, hence absent in K18, might also participate in the formation of protein/phospholipid complexes. The involvement of additional sequences would explain why MBD-K18 is unable to form protein/phospholipid complexes, whereas MBD-Tau can form such complexes, although to a much lesser extent than WT Tau. It is possible that the N-terminal domain, which has been reported to bind to membranes in vivo^12^, contains the additional structural determinants that are involved in modulating the capacity of Tau to form protein/phospholipids complexes.

The identification of mutations that disrupt membrane binding without affecting fibril formation provides a unique opportunity to assess the contribution of membrane binding to Tau aggregation, cell-to-cell propagation and toxicity in cells and in vivo. To assess whether the mutations disrupting membrane binding affect interaction of Tau with neuronal membranes and/or its uptake by neurons, we compared the uptake of monomers, monomer/phospholipid mixtures and fibrillar forms of WT and MBD mutants of Tau and K18 when added to primary hippocampal neurons. We found that primary neurons take up the MBD forms of Tau and K18 with substantially lower efficiency than the WT proteins. In the case of the monomeric proteins, only after 3 days did we observed a low level of diffuse and punctate staining of the cells, as well as some membrane staining for Tau (Supplementary Fig. 12a). Moreover, the MBD mutant-derived fibrils or the MBD mutants mixed with BPS vesicles were hardly observed inside the cells or bound to the neuronal plasma membranes (Supplementary Fig. 12b, c). These data strongly suggest that the MBD mutants are deficient in their ability to form protein/phospholipid complexes and to interact with neuronal membranes.

### Tau complexes are toxic to primary hippocampal neurons

**Fig. 8:**
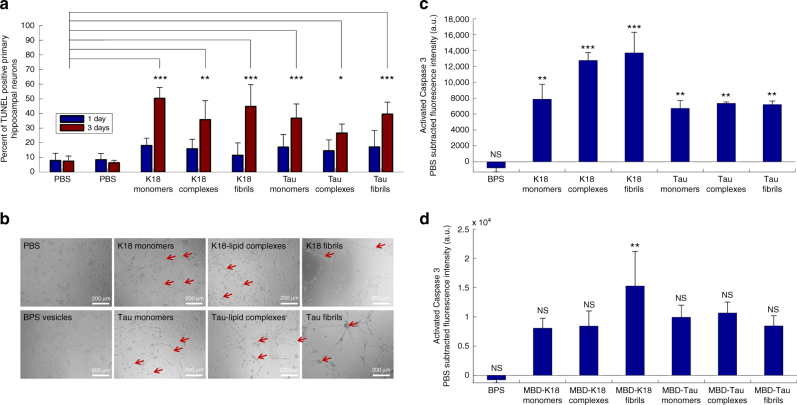
Tau and K18 protein/phospholipid complexes, fibrils, and monomers are toxic to hippocampal neurons and exert their toxicity through different mechanisms. Hippocampal mouse primary neurons were treated with PBS, BPS vesicles (controls), 3 µM Tau or K18 monomers, protein/phospholipid complexes or sonicated fibrils and fixed at 1 or 3 days post treatment. **a** Apoptotic cells were stained using the TUNEL assay, the neuronal population was stained using NeuN, and the nucleus was stained using DAPI. The percentage of apoptotic neurons [(TUNEL + and NeuN + cells)/NeuN + cells] was quantified as described in the methods. No statistical significance was observed at 1 day, whereas all species were significantly toxic at 3 days. Bars are mean ± S.D. **b** Tau and K18 monomers, fibrils, and protein/phospholipid complexes exert different effects on primary hippocampal neuron morphology. At 3 days, the PBS-treated and BPS-treated neurons present a healthy neuronal morphology with round cell bodies and extended neurites (left panels). Neurons treated with monomeric Tau and K18 show many condensed neurons (second left panels, arrows). Neurons treated with K18 or Tau fibrils clumped together, linked by bundles of neurites (second right panels, arrows). Tau/phospholipid complexes presented both condensed and clumped cell bodies, with bundled neurites (right bottom panel, arrows). K18/phospholipid complexes induced condensation of cell bodies and shrinkage of the neurites (right top panel, arrows). **c** Apoptotic cell death was confirmed using the CaspaTag fluorescein caspase 3 activity kit (Axxora) as described in the methods section. All species exhibited significant toxicity at 3 days post treatment. Bars are mean ± S.E.M. **d** The level of apoptosis induced by MBD-Tau and MBD-K18 species was assessed using the caspase 3 activity kit as described in the methods. The level of toxicity was higher than that of the BPS-treated neurons, but toxicity was only significant in the case of the MBD-K18 fibrils. Bars are mean ± S.E.M. (*p < 0.05, **p < 0.01, ***p < 0.001)

We studied the toxic properties of Tau and K18 protein/phospholipid complexes relative to those of monomer and fibrillar species added exogenously to neuronal primary culture by evaluating cell death as a function of time using the terminal deoxynucleotidyltransferase dUTP nick end labeling (TUNEL) detection assay for apoptotic cells^31^. Although no significant toxicity was observed at 1 day post-treatment, the neuronal population (identified as positive for NeuN, a specific neuronal marker), was severely affected by all the species at 3 days (Fig. 8a, b). The activation of apoptotic pathways was confirmed using a caspase 3 activity assay (Fig. 8c). We consistently observed that the K18 species exhibited more toxicity than the Tau species. The same tendency was also observed with the TUNEL assay, but to a lower extent. Moreover, the fibrils tended to be more toxic than the Tau and K18 protein/phospholipid complexes, which were more toxic than the monomers, especially in the case of K18. These differences would be expected between species inducing apoptosis through different mechanisms. However, such differences were not observed with the TUNEL assay. This discrepancy could result from the fact that the TUNEL assay was neuron-specific (only NeuN-positive cells were considered in the analysis), while the Caspase 3 activity assay included all cell types (i.e., both neuronal and glial cells present in our primary culture). A careful evaluation of the neurons post-treatment using well-characterized preparations of Tau monomers, protein/phospholipid complexes and fibrils (Fig. 8b) revealed important differences between the morphological effects of the treatments. While neurons treated with phosphate-buffered saline (PBS) and BPS presented a healthy neuronal morphology with well-defined, round cell bodies and extended neurites, neurons treated with monomeric Tau and K18 showed many condensed neurons (arrows in Fig. 8b). Conversely, neurons treated with K18 or Tau fibrils clumped together, linked by bundles of neurites. Tau/phospholipid complexes presented both condensed and clumped cell bodies, with bundled neurites. Finally, K18/phospholipid complexes induced condensation of cell bodies as well as shrinkage of the neurites. The fact that the different Tau species exhibited different degrees of internalization and induce distinct morphological phenotypes suggests that they could induce toxicity through distinct mechanisms.

To determine whether the MBD mutations influence Tau toxicity, we assessed the level of apoptosis induced by MBD-Tau and MBD-K18 monomers, monomers mixed with BPS vesicles and fibrils using the caspase 3 activity assay (Fig. 8d). Although we observed higher toxicity compared to the BPS-treated neurons, the level of toxicity was not significant, as determined using a post hoc Dunnett’s multicomparison test, except for the MBD-K18 fibrils. These results are in agreement with the uptake experiments and suggest that the reduced interaction of the mutants with the neuronal membrane and the resulting reduction in cellular uptake leads to lower levels of apoptotic cell death.

## Discussion

Growing evidence suggests that membranes play important roles in the nucleation and growth of fibrils formed by Tau and other amyloid proteins. However, the exact mechanisms by which Tau interacts with and/or remodels membranes remain elusive. Here, we report a mode of Tau interactions with negatively charged vesicles or vesicles that resemble the lipid composition of native neuronal membranes that leads to the formation of highly ordered, stable and toxic Tau/phospholipid complexes. Tau isoforms lacking the R2 repeat (0N3R and the K19 fragment) were deficient in forming protein/phospholipid complexes, suggesting that the R2 repeat plays an important role in the formation and stabilization of the Tau/phospholipid complexes, while the N-terminal inserts do not appear to be essential for the formation of Tau/phospholipid complexes. Although the formation of Tau oligomers in vitro has been previously reported by several groups^32–37^, we report here a previously undescribed oligomeric complex composed of both Tau and phospholipids.

Several groups have described the structural properties of fibrillar forms of Tau and the structure of Tau fragments using NMR and/or crystallography^38–40^. However, to date no structural studies have been performed on Tau oligomers. It is notable that the regions delineated here as forming the oligomer core (Fig. 5g) correspond quite closely to those that form the β-sheet core of mature filaments comprised of Tau K18^41^. In addition, the PHF6 and PHF6* motifs were shown to mediate inter-molecular contacts in heparin-induced Tau oligomers^42^, suggesting a potentially general role for these regions in oligomer formation. Surprisingly, carbon chemical shifts for oligomer core signals originating from outside PHF6 (i.e., the PHF6* motif, and the ~9 residues following each hexapeptide motif) indicate a lack of any secondary structure preference. These regions, while part of the rigid oligomer core, thus do not adopt β-strand, or other, secondary structure. These findings suggest that, although subdomains of R2 and R3 C-terminal from the hexapeptides fold into α-helices upon binding to membranes, as previously described^16,17,20^, in the fully formed K18/phospholipid complexes, these regions, while part of the oligomeric core, are devoid of any secondary structure. It is plausible that the binding to membranes induces a transient α-helical state that is, however, not preserved in the oligomer core. These data are consistent with the notion that our Tau/phospholipid complexes represent a precursor to more mature disease-linked fibrillar species, but also raises interesting questions regarding the interactions that stabilize the oligomer core in apparently rigid conformations that are nevertheless largely devoid of secondary structure.

On the basis of these findings, we hypothesize that disrupting the amphipathic nature of these α-helices may prevent the initial membrane binding and therefore also the subsequent formation of protein/phospholipid complexes, even though the final Tau/phospholipid complexes do not contain α-helices. To test this hypothesis, we generated MBD mutants and showed that disrupting the amphipathic character of the R2, R3 α-helices and the PHF6 β-sheet within Tau reduced membrane binding, abrogated protein/phospholipid complex formation and cellular uptake by hippocampal primary neurons, and lowered Tau toxicity.

Although Tau is an intracellular protein, growing evidence suggests that pathological Tau is secreted from neurons^43–45^ and is then taken up by neighboring neurons and other cells^46–48^. The release, secretion and uptake of Tau are thought to contribute to the mechanisms by which Tau pathology spreads from one cell to another and throughout the brain. However, the exact forms of Tau that mediate its cell-to-cell propagation and pathology spreading in AD, and the mechanisms by which they are transported between neurons remain unknown. To determine if the Tau/phospholipid complexes could be involved in mediating Tau cell-to-cell transfer and/or toxicity, we investigated the uptake and toxicity of these complexes in primary hippocampal neurons in comparison to monomeric and fibrillar forms of Tau and K18. Our findings demonstrate that the Tau and K18 protein/phospholipid complexes are taken up by neurons through the endocytic pathway, but appear to escape from it, as they co-localized with an early endosome marker, but not with a late endosome and lysosome marker. These data are in agreement with previously published studies reporting the uptake of exogenous oligomeric Tau species^47,49^ through endocytosis^33^.

Interestingly, we observed that monomeric, fibrillar and protein/phospholipid Tau complexes all induce neuronal apoptosis when exogenously added to hippocampal primary neurons. The Tau and K18 protein/phospholipid complexes showed similar overall toxicity as the mature Tau fibrils, but seem to exert their toxic effects through a distinct mechanism. Although the toxicity of the Tau monomers was initially surprising, a careful and extensive review of the literature revealed that similar observations have been reported before^50–52^. A recent study by Mirbaha et al.^53^ suggested that monomeric Tau exists in an ensemble of conformations, some of which are seed-competent and capable of templating Tau aggregation and fibril formation in vitro and in cells. Furthermore, our observation that monomeric extracellular Tau itself is also toxic to primary neurons suggests that physiological and/or pathological transfer of Tau between neurons or neurons and other cell types must occur under conditions in which Tau monomers are chaperoned and/or protected from direct interactions with neurons. The notion that different Tau and K18 species have different mechanisms of toxicity is supported by the studies of many groups that have attributed different molecular mechanisms of toxicity to each of the Tau and K18 species^54^. Monomeric Tau has been shown to induce toxicity through muscarinic receptor-induced liberation of Ca^2+^ ^50,51^, whereas oligomeric Tau might exert its toxicity through deleterious interaction with the cell membrane^35^, facilitated endocytosis^55^, pore formation^35^, and/or deleterious interactions with neuronal spines^56^. The question whether Tau fibrils are toxic^50^, protective or innocent^32,56^ remains controversial^57^, although there is strong evidence that exogenously added synthetic Tau fibrils can template de novo aggregates in cells and mouse models^58–60^. Altogether, our results suggest that, although the overall level of apoptosis induced by the different species is similar, the fact that the different Tau and K18 species exhibit different modes of interactions with neurons, different degree of internalization and subcellular localization, and induce distinct morphological phenotypes, suggests that they could be exerting their toxic effects through distinct mechanisms.

**Fig. 9:**
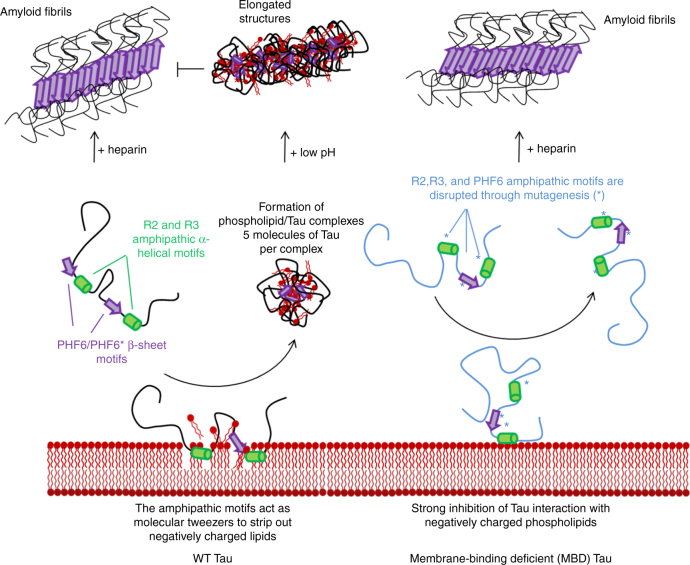
Schematic depiction of our working model that summarizes our findings for the interaction of Tau with negatively charged phospholipids. Tau forms fibrils readily in the presence of heparin, and Tau monomers interact with vesicles containing negatively charged phospholipids, potentially though the formation of short amphipathic α-helical (green) and β-sheet (purple) motifs^16,17,20^. Subsequently, phospholipid molecules are segregated into highly stable protein/phospholipid complexes, possibly through a tweezers mechanism (left). The core of these protein/phospholipid complexes consists of PHF6 and PHF6*, and regions C-terminal to these two motifs, whereby only the PHF6 is folded into a β-sheet, while the rest of the core remains unfolded. Based on these findings, mutants were designed that retain the ability to from fibrils in the presence of heparin, but no longer interact with phospholipids (right) to form protein/phospholipid complexes, thus providing powerful tools to investigate how membrane binding affects the normal and pathological functions of Tau

A working model that summarizes our main findings is presented in Fig. 9. We propose that the repeat peptides R2 and R3, as well as the PHF6 motif, which are all individually capable of remodeling the membrane, could, in the context of K18 and full-length Tau, act as molecular tweezers that could extract phospholipids from the membrane, leading to (1) membrane disruption, which might lead to cytotoxicity and (2) the formation of protein/phospholipid complexes, which themselves are rapidly internalized by neurons and possibly other cell types.

Although extremely stable, the Tau/phospholipid complexes can convert into elongated structures and higher-order assemblies under conditions that proteins experience as they go through the endosomal-lysosomal pathway (i.e., acidic pH). The drastic change in the morphology of the Tau/phospholipid complexes at slightly acidic pH suggests a possible mechanism by which these complexes could serve as precursors for nucleating Tau aggregation upon internalization by neurons. This hypothesis is consistent with the NMR data pointing to structural similarities between the Tau/phospholipid complexes and Tau fibrils and with the fact that these complexes are recognized by the MC-1 antibody, a well-established marker of pathological Tau conformations. However, the structural and morphological properties of these assemblies do not resemble those of Tau oligomers or fibrils, suggesting that changes in pH may be sufficient to initiate the polymerization of Tau, but the transition to β-sheet-rich fibrils may require additional secondary structure changes that may be accessible in the cellular environment. Together, these findings suggest that Tau/phospholipid complexes could provide a mechanism for stabilizing Tau and may play important roles in the trafficking, toxicity and/or cell-to-cell propagation of Tau in vivo.

Our findings point toward a novel form of Tau protein/phospholipid complexes that might be part of a membrane-dependent mechanism that regulates Tau structure, oligomerization, toxicity and possibly its normal and aberrant trafficking between and within neurons. Further studies are underway to determine if such Tau/phospholipid complexes occur in vivo and whether targeting these complexes attenuates Tau aggregation, toxicity, and pathology spreading. Finally, our work underscores the critical importance of a better understanding of the mechanisms as well as the molecular and structural determinants of Tau interactions with membranes and how these interactions affect Tau structure, function(s), trafficking and cell-to-cell transfer in health and disease. In this regard, the design and validation of novel membrane binding-deficient mutants that inhibit the ability of Tau to interact with membranes and prevent the formation of Tau/phospholipid complexes, such as those described in this work, provides powerful tools to elucidate the contribution of Tau-membrane interactions to the regulation of Tau cellular properties and neurotoxicity.

## Methods

### Antibodies and phospholipids and peptides

Monoclonal mouse anti-human Tau antibody (Tau13 cat n° ab19030, dilution 1:5000) and polyclonal rabbit anti-EEA1 (cat n° ab2900, dilution 1:50) were purchased from Abcam, monoclonal mouse anti-NeuN antibody from EMD Millipore (cat n° MAB377, dilution 1:100), polyclonal rabbit anti-MAP2 antibody from Synaptic Systems (cat n° 188002, dilution 1:500), and monoclonal mouse anti-LAMP1 from BD Transduction lab (cat n° 611043, dilution 1:50). MC-1 antibody (dilution 1:500), described in ref. ^25^ was a kind gift from Prof. Peter Davies (Department of Pathology, Albert Einstein College of Medicine). Polyclonal rabbit anti-total Tau antibody (dilution 1:5000) was generated in house. Fluorescently labeled secondary antibodies (donkey anti-mouse Alexa 647, cat n° A31571; donkey anti-rabbit Alexa 488, cat n° A21206; donkey anti-rabbit Alexa 647, cat n° A31573; dilutions 1:800) were purchased from Molecular Probes (Life Technologies).

Phosphatidylserine isolated from porcine brain (BPS), 1,2-dioleoyl-sn-glycero-3-phosphocholine (DOPC), porcine brain total lipid extract (TBE), and fluorescently labeled 1-oleoyl-2-{6-[(7-nitro-2-1,3-benzoxadiazol-4-yl)amino]hexanoyl}-sn-glycero-3-phosphoserine (fPS) were purchased from Avanti Polar Lipids, Inc.

The PHF6 peptide (306-VQIVYK-311) was synthesized. Due to the synthesis strategy, the peptide used in this study contains an extra glycine residue at the C terminus (VQIVYKG). The R1, R2, R3, and R4 peptides (244-QTAPVPMPDLKNVKSKIGSTENLKHQ-269, 275-VQIINKKLDLSNVQSKCGSKDNIKHV-300, 306-VQIVYKPVDLSKVTSKCGSLGNIHHK-331, 337-VEVKSEKLDFKDRVQSKIGSLDNITHV-363) corresponding to the four individual microtubule-binding repeats were purchased from CS Bio Co.

### DNA constructs and mutagenesis

The K18 fragment was synthesized by GeneArt Gene Synthesis (Life Technologies) and cloned into the pT7-7 expression vector.

Codon-optimized DNA sequence: 5′-ATGCAGACCGCACCGgTTCCGATGCCGGATCTGAAA AATGTTAAAAGCAAAATTGGCAGCACCGAAAATCTGAAACATCAGCCTGGTGGTGGTAAAGTGCAGATTATTAATAAAAAACTGGATCTGAGCAATGTGCAGAGCAAATGTGGTAGCAAAGATAATATTAAACATGTTCCGGGTGGTGGTAGCGTTCAGATTGTTTATAAACCGGTGGATCTGAGCAAAGTTACCAGCAAATGTGGTAGCCTGGGCAATATTCATCATAAACCGGGTGGTGGCCAGGTTGAAGTTAAAAGCGAAAAACTGGATTTTAAAGATCGCGTGCA GAGCAAAATTGGTTCCCTGGATAATATTACCCATGTTCCGGGTGGTGGCAATAAAATTGAATAA-3′

Single-site mutagenesis was performed with Tau441 in pET-15b and K18 in pT7-7 using the QuikChange Site-Directed Mutagenesis Kit (Stratagene).

List of primers (forward): K18 V287E: 5′-CTGGATCTGAGCAATGAACAGAGCAAATGTGGTAGC-3′; K18 V318E: 5′-CCGGTGGATCTGAGCAAAGAAACCAGCAAATGTGGTAGC-3′; K18 I308E: 5′-GTGGTGGTAGCGTTCAGGAGGTTTATAAACCGGTGG-3′; Tau V287E: 5′-CTGGATCTTAGCAACGAGCAGTCCAAGTGTGGC-3′; Tau V318E: 5′-GTTGATCTGAGCAAGGAGACCTCCAAGTGTGG-3′; Tau K311A: 5′-GTGTGCAAATAGTCTACGCACCAGTTGATCTGA-3′.

### Protein expression and purification

Tau isoforms 2N4R, 0N4R, and 0N3R in pET-15b, and K18 and K19 in pT7-7 were expressed in *Escherichia coli* strain BL21 [One Shot BL21 (DE3), Life Technologies]. K19 and Tau 0N3R were purified as described in^61^. The cells were lysed by sonication in a solution containing 3 M urea in 10 mM Tris, pH 8.0, 1 mM EDTA, 1 mM DTT, 1 mM PMSF and followed by ultracentrifugation at 192,839×*g* in a Beckman ultracentrifuge using a Ti 50.2 rotor. The supernatant was further treated with streptomycin sulphate, followed by dialysis against 25 mM Tris, pH 8.0, 20 mM NaCl, 1 mM EDTA, and 1 mM DTT and applied to a cation-exchange column and eluted with a NaCl gradient. Fractions containing the protein were pooled, dialyzed against 5% acetic acid, and purified on either a C18 (K19) or C4 (0N3R Tau) reverse-phase HPLC column using an acetonitrile gradient with 1% trifluoroacetic acid. The purified protein was lyophilized and stored at −20 °C. Purity was verified by SDS-PAGE. The purification protocol for Tau 2N4R and 0N4R was adapted from ref. ^62^. The cells were pelleted and broken by sonication in lysis buffer (3 M urea in 10 mM MES, pH 6.5, 1 mM DTT, 1 mM EDTA, 1 mM PMSF). After centrifugation at 150,000×*g* for 1 h at 4 °C, 1% (w/V) of streptomycin sulfate was added to the supernatant, and the solution was stirred for 90 min at 4 °C. After centrifugation at 27,000×*g* for 1 h at 4 °C, the supernatant was dialyzed overnight at 4 °C in ion exchange (IEX) buffer A (10 mM MES, pH 6.5, 20 mM NaCl, 1 mM DTT, 1 mM EDTA). The supernatant was filtered and loaded on a cation-exchange column (MonoS, GE Healthcare), and the protein was eluted using a salt gradient (increasing the NaCl concentration of IEX buffer A from 20 mM to 1 M NaCl over 20 column volumes). Fractions containing the proteins were dialyzed overnight against acetic buffer (5% acetic acid in water) and loaded on a reverse-phase HPLC C4 column (PROTO 300 C4 10 µm, Higgins Analytical; buffer A: 0.1% TFA in water, buffer B: 0.1% TFA in acetonitrile), and the protein was eluted using a gradient from 30 to 40% buffer B over 40 min (15 ml min^−1^). K18 in pT7-7 was purified following a protocol adapted from ref. ^61^. Briefly, cells were broken by sonication in IEX buffer B (10 mM HEPES, pH 6.5, 1 mM MgCl_2_, 20 mM NaCl, 1 mM DTT, 1 mM PMSF). After centrifugation at 40,000×*g* for 30 min, the supernatant was boiled until the solution became cloudy (~5 min) and centrifuged again for 30 min. The supernatant was filtered and loaded on a cation-exchange column (MonoS, GE Healthcare), and the protein was eluted using a salt gradient (increasing the NaCl concentration of IEX buffer B from 20 mM to 1 M NaCl over 20 column volumes). Fractions containing the K18 fragment were immediately loaded on a reverse-phase HPLC C4 column (PROTO 300 C4 10 µm, Higgins Analytical; buffer A: 0.1% TFA in water, buffer B: 0.1% TFA in acetonitrile), and the protein was eluted using a gradient from 30 to 40% buffer B over 40 min (15 ml min^−1^). Isotopically labeled K18 was expressed in a 13C/15N-labeled minimal medium and purified as described above.

### Oregon Green maleimide labeling of K18

Recombinant K18 (5 mg) was dissolved in 1.5 ml 200 mM Tris, pH 7.0. Four equivalents of Oregon Green (OG) maleimide (0.7 mg) (Life Technologies) were added to the protein solution and incubated for 2 h at RT, followed by SEC on a PD10 column (GE healthcare) to remove excess dye. Fractions containing labeled protein, identified by electrospray ionization mass spectrometry and SDS-PAGE, were combined and lyophilized.

### TAMRA labeling of WT Tau and K18 and MBD mutants

Recombinant MBD-K18 and MBD-Tau (2 mg) were dissolved in 1 ml 10 mM phosphate buffer pH 7.4, 50 mM NaF. Two equivalents of tetramethylrhodamine-5-maleimide (TAMRA) (Sigma) were added to the protein solution and incubated for 15 min at RT (the level of cystein labeling was followed by ESI-MS). When fully reacted, excess dye was removed by ethanol precipitation. Briefly, 14 ml of 100% ethanol was added to the reaction and placed at −80 °C overnight. The tube was centrifuged at 4000×*g* for 30 min and the supernatant containing unreacted dye was discarded. To remove any remaining unbound dye, another 10 ml of ice-cold 100% ethanol was added to the pellet, the tube was centrifuged again for 15 min at 4000×*g* and the supernatant was discarded. The pellet containing the labeled protein was dried and lyophilized.

### Preparation of heparin-induced Tau fibrils

The fibrils of WT Tau and K18 and MBD mutants were formed by incubating monomeric protein in 10 mM HEPES, pH 7.4, 100 mM NaCl, and 2.5 mM DTT with heparin sodium salt (Applichem GmbH) at a molar heparin:protein ratio of 1:4 under constant orbital agitation (1000 r.p.m., Peqlab, Thriller) for 72 h at 37 °C. When needed, free heparin was removed by subjecting the fibrils to filter-centrifugation and resuspending the fibrils trapped in the filter.

### Preparation of seeds-induced Tau fibrils

The seeds were prepared by sonicating heparin-induced fibrils for 10 min (ultrasonic cleaner, VWR). Seeds were snap-frozen in liquid nitrogen and stored at −80 °C until use. Seeded fibrils were prepared by incubating monomeric protein with 10% (molar) seeds under constant orbital agitation (1000 r.p.m., Peqlab, Thriller, Germany) for 72 h at 37 °C. To obtain a homogeneous distribution of short fibrils, the fibrils were broken down by sonication for 10 min in a bath sonicator (ultrasonic cleaner, VWR). The presence and morphology of the fibrils was assessed using Thioflavin T (ThT) fluorescence and EM.

### Vesicle formation

Vesicles were prepared using the extrusion method. Briefly, individual phospholipids or phospholipid mixtures in chloroform were dried using an argon stream to form a thin film on the wall of a glass vial. Potentially remaining chloroform was removed by placing the vial under vacuum overnight. The phospholipids were resolubilized in 10 mM HEPES, pH 7.4, 100 mM NaCl, and 2.5 mM DTT to the desired final concentration by sonication. The solution was then passed through an Avestin LiposoFast extruder *(*Avestin Inc.) (membrane pore size: 0.1 µM), and the size and homogeneity of the resulting vesicles were assessed by EM. For the preparation of fluorescently labeled vesicles, fluorescent, and non-fluorescent phospholipids in chloroform were mixed at a molar ratio of 1:99 prior to the initial drying step.

### Specimen preparation for electron microscopy

The samples (3.5 μl) were applied onto glow-discharged Formvar/carbon-coated 200-mesh copper grids (Electron Microscopy Sciences) for 1 min. The grids were blotted with filter paper, washed twice with ultrapure water and once with staining solution (uranyl formate 0.7% (w/V)), and then stained with uranyl formate for 30 s. The grids were blotted and dried.

### Electron microscopy imaging

The specimens were inspected with two electron microscopes: a Philips CM10 operated at 100 kV and equipped with a tungsten filament and a Gatan 2K × 2K CCD camera, and a Tecnai Spirit BioTWIN operated at 80 kV and equipped with an LaB_6_ filament and a 4K × 4K FEI Eagle CCD camera. For single-particle analysis, a Tecnai T12 electron microscope operated at 120 kV and equipped with an LaB_6_ filament and a Gatan 4K × 4K CCD camera was used to collect images using low-dose procedures and a defocus of 1.5 μm.

### Single-particle averaging

For K18 12,000 particles and for Tau 8100 particles were manually selected using Boxer^63^ and classified with the *K*-means algorithm into 200 classes using the SPIDER software package^64^. Classes that produced averages showing Tau particles with a radius ranging from 100 to 110 Å (~4850 particles) or averages showing K18 particles with a radius ranging from 81 to 97 Å (~4000) were combined and classified again, specifying 50 output classes. These averages are shown in Fig. 3b.

### Vesicle sedimentation assay

The centrifuge tubes were filled with 2.8 ml of 60% (w/V) sucrose, which was then overlayed with 0.4 ml of 10% sucrose. The sample (0.3 ml) at a molar phospholipid:protein ratio of 100:1 was applied to the top of the step gradient and centrifuged at 200,000×*g* for 90 min at RT using a Beckmann swinging bucket SW41 rotor. Fractions (0.2 ml) were collected from top to bottom and analyzed by EM and SDS-PAGE.

### Preparation of mixed protein/phospholipid complexes

The protein/phospholipid complexes were prepared by incubating BPS vesicles with K18 or Tau at a molar protein:phospholipid ratio of 1:20 in 10 mM HEPES, pH 7.4, 100 mM NaCl, and 2.5 mM DTT for 48 h at 37 °C. When specified, the resulting protein/phospholipid complexes were separated from remaining vesicles and soluble protein by SEC using a Superose 6 column (GE Healthcare).

### NativePAGE

Acrylamide gels (7.5%) were prepared without SDS and run at 120 V for 2 h in 1X NativePAGE running buffer (Life Technologies) for the anodic compartment and 1X NativePAGE buffer supplemented with 1X cathodic buffer additive (Life Technologies) for the cathodic compartment. The gels were destained in 40% methanol and 10% acetic acid.

### Circular dichroism spectroscopy

CD spectra were recorded on a Jasco J-815 CD spectrometer operated at 20 °C. To minimize buffer absorption, the buffer was modified to 10 mM phosphate buffer, pH 7.4, with 50 mM NaF and 0.5 mM DTT. CD spectra were acquired from 195 to 250 nm at a scan rate of 50 nm min^−1^ and in increments of 0.2 nm. For each sample, three spectra were averaged and smoothed using binomial approximation. For protein and protein/phospholipid samples (Tau and K18, WT and MBD mutants), the protein concentration was adjusted to 10 µM (when applicable, the molar protein:phospholipid ratio was 1:100 or 1:20). For peptide and peptide/phospholipid samples, the peptide concentration was adjusted to 200 µM (when applicable, the molar peptide:phospholipid ratio was 1:1). To determine melting temperatures, the temperature of the CD cuvette was varied from 10 to 90 °C, and spectra were recorded every 10 °C over a range of 195–250 nm with a scan rate of 50 nm min^−1^ and in increments of 0.2 nm. Five spectra were averaged and smoothed using binomial approximation.

### Nuclear magnetic resonance spectroscopy

Solid-state NMR spectra of [U-13C/15N]-labeled K18 protein/phospholipid complexes were obtained using 3.2-mm triple-resonance (1H, 13C, and 15N) probe heads on a Bruker AVANCE II 750 MHz/17.6 T spectrometer at the New York Structural Biology Center (NYSBC). Two independent samples gave rise to highly reproducible DARR spectra. 13C−13C magnetization transfer during the mixing time was accomplished using the dipolar-assisted rotational resonance (DARR) condition^65^ at a sample temperature of about 278 K and a magic-angle spinning speed of 13 kHz. An initial ramped cross-polarization element^66,67^ was used to transfer the magnetization from 1H to 13C with contact times of 900 μs and 1000 μs for the 20-ms and 150-ms DARR spectra, respectively. TPPM high-power proton decoupling^68^ was applied during evolution and detection periods with radiofrequency amplitudes of 92 kHz and 71.4 kHz for the 20-ms and 150-ms DARR spectra, respectively. Carbon chemical shifts were calibrated with adamantine as an external reference^69^. All spectra were processed with Topspin and analyzed in SPARKY version 3.1 (T.D. Goddard and D.G. Kneller, University of California).

Solution-state HSQC spectra were collected on Bruker AVANCE 800 (NYSBC) or 600 MHz (Weill Cornell) spectrometers at a sample temperature of 283 K for monomeric samples and for protein/phospholipid complexes preparations prior to centrifugation of the samples to generate the solid-state NMR samples. Resonance assignments were annotated as reported previously^16^. Oligomer-to-monomer intensity ratios were arbitrarily normalized so that values at the very N and C termini were close to one.

### Multi-angle light scattering

Multi-angle light scattering (MALS) was performed on an Agilent Technologies series 1200 instrument coupled to a DAWN 8+ multi-angle light scattering detector (Wyatt Technology Corporation) and an Optilab rEX refractive index detector (Wyatt Technology Corporation). Protein/phospholipid complexes prepared as described above were run over a Superose 6 column (GE Healthcare) at a flow rate of 0.4 ml min^−1^. Normalization, alignment and adjustment for band broadening were performed using bovine serum albumin (BSA) (run immediately prior to the sample). The following parameters were used for the analysis: K18 extinction coefficient: 110 [ml g^−1^ cm^−1^], Tau extinction coefficient: 162 [ml g^−1^ cm^−1^], protein dn/dc: 0.185 [ml g^−1^] (accepted value), BPS dn/dc: 0.16 [ml g^−1^] (accepted value for phospholipids), BPS extinction coefficient: 0.7 (measured). The data analysis was performed using the Astra 5.3 analysis software program (Wyatt Technology Corporation).

### Sedimentation assay

Taking advantage of the fact that high-speed centrifugation sediments fibrils but not monomeric or soluble oligomeric species, the sedimentation assay makes it possible to assess the ratio of fibrilized:soluble protein. BPS-induced Tau and K18 fibrils (20 µl) were pelleted by ultracentrifugation at 200,000×*g* for 2 h at 4 °C. Both the supernatant and the resuspended pellet were run on 15% SDS-PAGE gels.

### Co-sedimentation assay

Co-sedimentation was used to assess initial binding of protein to vesicles. Tau or K18 (10 µM) were mixed with BPS vesicles (200 µM) to a total volume of 20 µl and immediately centrifuged at 200,000×*g* for 2 h at 4 °C. At this speed, the vesicles pellet, and protein bound to them co-sediment with the vesicles. Both the supernatant, containing unbound protein, and the resuspended pellet, containing vesicles with bound protein, were run on 15% SDS-PAGE gels.

### Thioflavin T fluorescence measurements

Thioflavin T (ThT) fluorescence reading (excitation wavelength of 450 nm, emission wavelength of 485 nm) was performed in triplicate with a ThT concentration of 60 μM and a peptide concentration of 60 μM in 50 mM glycine, pH 8.5, using a Bucher Analyst AD plate reader.

### Mouse primary hippocampal neuron culture

Hippocampi from P0 WT mice (C57BL/6JRccHsd, Harlan laboratories) were dissociated and triturated in papain-containing medium. After centrifugation at 400×*g* for 2 min, neurons were plated in MEM supplemented with 10% fetal calf serum (FCS) on Cultrex poly-L-lysine (Trevigen)-coated coverslips at 1.5 × 10^5^ cells ml^−1^ (6 well-plates, 3 ml per well). After 4 h, medium was replaced with Neurobasal/B27 medium, and neurons were grown for 6 days before being treated with 2.3 µM cytosine β-*D*-arabinofuranoside to inhibit glial cell growth. Treatments with extracellular proteins were performed 2 weeks after plating.

### Immunocytochemistry

Hippocampal primary neurons were seeded on coverslips coated with Cultrex poly-L-lysine and treated with 1 µM Tau or Oregon Green-labeled K18 in the form of monomers, protein/phospholipid complexes or fibrils for 1 or 3 days, after which the coverslips were fixed in 4% paraformaldehyde for 20 min at 4 °C. After blocking with 3% BSA and 0.1% Triton X-100 in PBS (PBS-BSA-T) for 30 min at RT, the coverslips were incubated with primary antibodies (mouse anti-Tau (Tau13), dilution 1:5000, and/or rabbit anti-MAP2, dilution 1:500) for 2 h at RT. The neurons were rinsed five times with PBS-BSA-T, incubated with the secondary antibodies (donkey anti-mouse Alexa 647, donkey anti-rabbit IgG Alexa 488, donkey anti-rabbit Alexa 647, dilution 1:800) and DAPI (1:1000) for 1 h at RT, then washed five times with PBS-BSA-T and kept in PBS until mounting in polyvinyl alcohol mounting medium supplemented with anti-fading DABCO reagent. The coverslips were examined using a confocal laser-scanning microscope (LSM 700, Zeiss Microscopy) with a ×40 objective and analyzed using the Zen software (Zeiss Microscopy). For co-staining with endolysosomal markers (EEA1 and LAMP1), PBS-BSA-T was replaced by 0.02% saponin (Sigma-Aldrich), 3% BSA in PBS.

### Quantification of apoptotic death using the TUNEL assay

The DNA fragmentation arising from apoptosis was detected using the terminal deoxynucleotidyltransferase-mediated dUTP-biotin nick end labeling (TUNEL) method^31^. Mouse primary hippocampal neurons seeded on coverslips coated with Cultrex poly-L-lysine were treated with 3 µM of Tau or K18 in the form of momomers, protein/phospholipid complexes or sonicated fibrils for 1 or 3 days, after which the coverslips were fixed in 4% paraformaldehyde for 20 min at RT. Following permeabilization with 0.1% Triton X-100 and 0.1% sodium citrate for 2 min on ice, coverslips were washed five times with PBS, pH 7.4, and incubated with the TUNEL reaction mix for 1 h at 37 °C, as per manufacturer’s instructions (In Situ Cell Death Detection kit, Roche). Following five PBS rinses, blocking was performed by incubating the coverslips in PBS-BSA-T for 30 min at RT. The coverslips were then incubated for 2 h with mouse anti-NeuN primary antibody (1:100) at RT, rinsed five times with PBS-BSA-T, and incubated for 1 h with the secondary antibody (donkey anti-mouse Alexa 647, 1:800) and 4′,6-diamidino-2-phenylindole (DAPI) (1:1000) at RT. The neurons were then washed five times with PBS-BSA-T and kept in PBS until mounting in polyvinyl alcohol mounting medium supplemented with the anti-fading DABCO reagent (Sigma-Aldrich). Coverslips were examined with a bright-field light microscope (Axioplan, Zeiss Microscopy) and analyzed using the Image J program (U.S. National Institutes of Health). The percentage of apoptotic neurons—[cells positive for NeuN and TUNEL]/[cells positive for NeuN] was quantified by counting three fields per condition with an average of 300 cells per field. The data shown represent the mean of four independent experiments performed in triplicate for each condition (bars are mean ± S.D.). A one-way ANOVA test followed by a Dunnett test was performed for cells treated with PBS versus cells treated with Tau or K18 species.

### Quantification of active caspase 3 in primary neurons

CaspaTag fluorescein caspase 3 activity kit (Axxora) was used to quantify the level of active caspase 3 in living cells. This kit uses fluorochrome peptide inhibitors of caspases, the FAM-DEVD-FMK, which passively enter cells and bind to the active form of effector caspase 3. Mouse primary hippocampal neurons seeded on black clear bottom 96-well plates coated with Cultrex poly-L-lysine were treated with 3 µM of Tau or K18 WT/MBD mutants in the form of monomers, protein/phospholipid complexes or sonicated fibrils for 3 days, after which primary neurons were washed three times with PBS and incubated for 30 min at 37  °C with FAM-DEVD-FMK as per manufacturer’s instructions. Fluorescein emission was quantified using a Tecan infinite M200 Pro plate reader (Tecan) with excitation and emission wavelengths of 487 and 519 nm, respectively. The data shown represent the mean of three independent experiments performed in triplicate for each condition (bars are mean ± S.E.M.). A one-way ANOVA test followed by a Dunnett test was performed for cells treated with PBS versus cells treated with Tau or K18 species.

### Statistics

The data from independent experiments show a normal distribution with a similar variance with *n* = 4 (TUNEL) or *n* = 3 (caspase 3 activity). To compare all the conditions between them, we performed a multicomparison test to assess the significance of the differences observed in our samples. The statistical analyses were performed using a one-side ANOVA test followed by a Dunnett post hoc test, as it allows pairwise comparisons of multiple treatment groups with a single control group (in our case PBS-treated control versus treated conditions). The data were regarded as statistically significant at *p*-value < 0.05. (*p < 0.05, **p < 0.01, ***p < 0.001).

### Data availability

The data are available from the corresponding author upon reasonable request.

## Electronic supplementary material

Supplementary Information
