# Supplementary material for: Discovery and characterization of stable and toxic Tau/phospholipid oligomeric complexes
Source: Nat Commun. 2017 Nov 22;8:1678. doi: 10.1038/s41467-017-01575-4 (PMC5698329; doi:10.1038/s41467-017-01575-4)

Supplementary Information

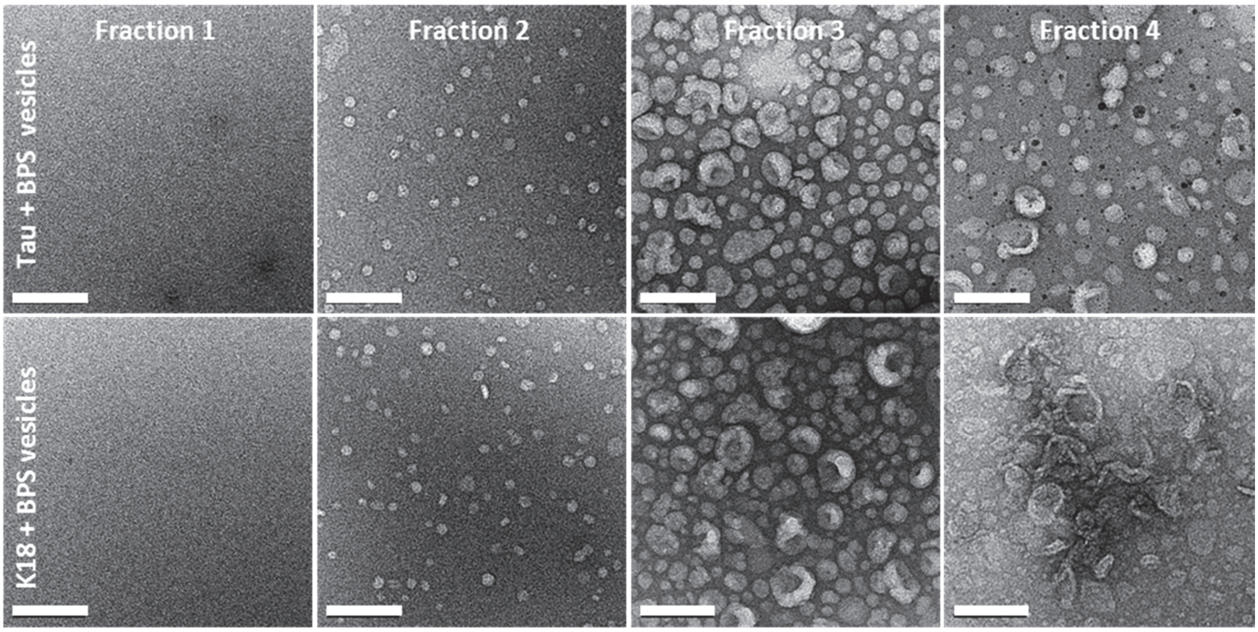

Supplementary Figure 1 | Analysis of vesicle sedimentation fractions.
EM analysis of vesicle sedimentation assay fractions (Fig. 1). Negative-stain EM images are shown for fractions 1 to 4 for Tau (top) and K18 (bottom) mixed with BPS vesicles at a molar protein:phospholipid ratio of 1:100. The scale bars are 100 nm.

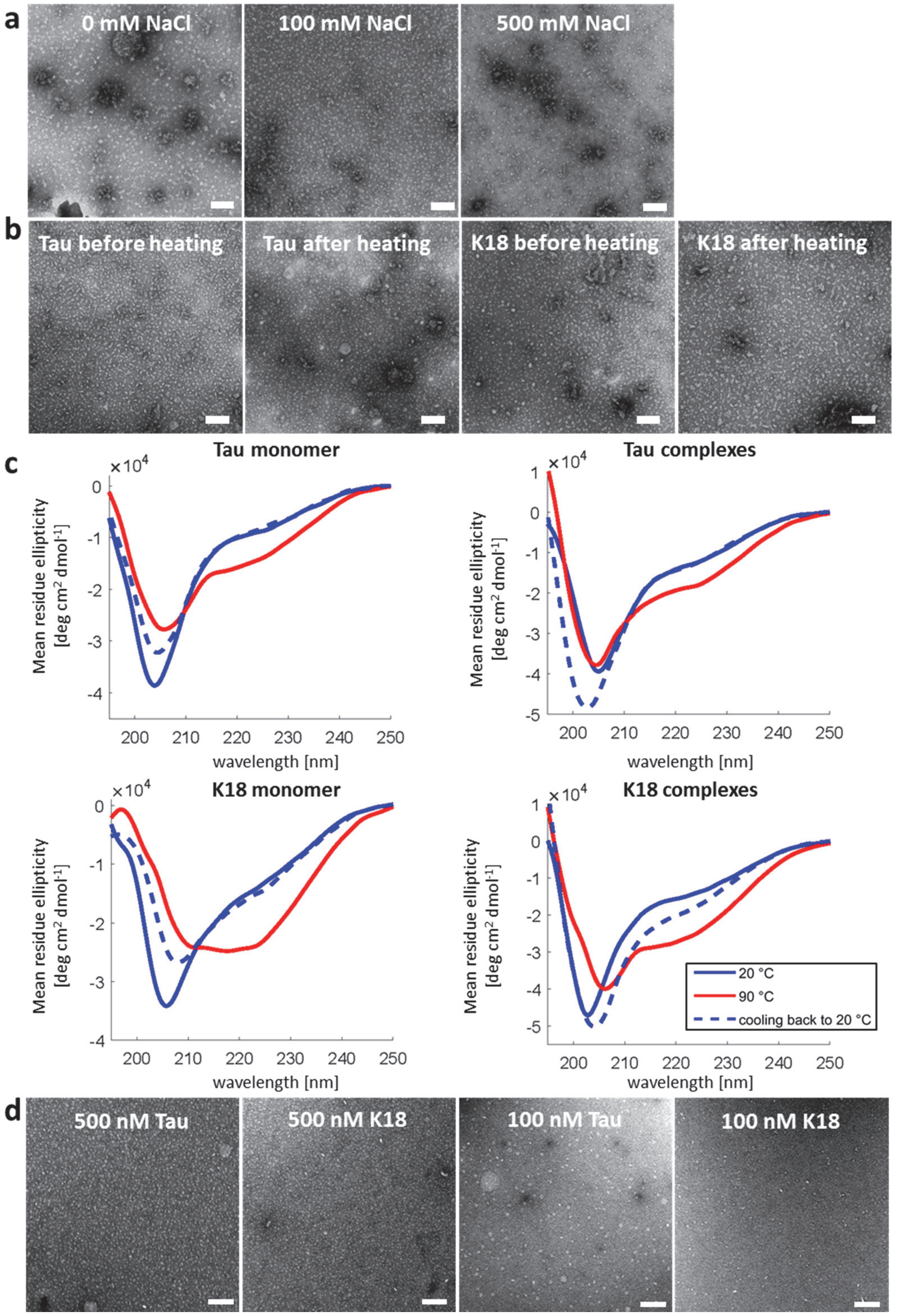

Supplementary Figure 2 | Stability of the Tau and K18 protein/phospholipid complexes.

(**a**) Tau/phospholipid complexes are stable in the presence of 0 and 500 mM NaCl. Negative-stain EM images of Tau/phospholipid complexes prepared under standard conditions using 100 mM NaCl (middle panel) and dialyzed either against 0 mM (left panel) or 500 mM NaCl (right panel), showing that the complexes are stable over a wide range of ionic strength conditions. The scale bars are 100 nm. (**b**) Tau and K18 protein/phospholipid complexes are stable at high temperature. Negative-stain EM images showing Tau and K18 protein/phospholipid complexes at room temperature and after heating the samples to 90°C. The EM images show that heating to 90°C has little effect on the appearance of protein/phospholipid complexes. The scale bars are 100 nm. (**c**) CD spectra of Tau and K18 monomers and protein/phospholipid complexes measured at 20°C (blue line), after heating to 90°C (red line) and after cooling back to 20°C

(blue dashed line). Both the monomeric proteins and protein/phospholipid complexes show a significant increase in secondary structure upon heating, and recover their initial spectral signature upon cooling down to 20°C. (**d**) Tau and K18 protein/phospholipid complexes are formed at sub-physiological Tau concentrations. Negative-stain EM images showing complexes of Tau and K18 with phospholipids at concentrations of 500 nM (left) and 100 nM (right), which are below the physiological Tau concentration of ~2 μM. The EM images show that the protein/phospholipid complexes form even at a concentration as low as 100 nM. The scale bars are 100 nm.

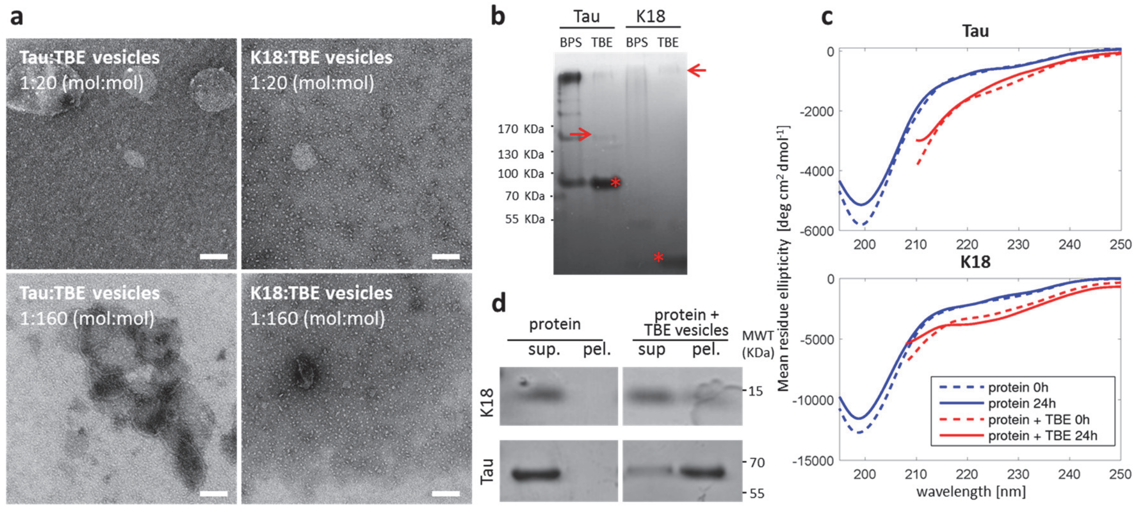

Supplementary Figure 3 | Tau and K18 form protein/phospholipid complexes in the presence of TBE vesicles.
(**a**) EM analysis of Tau (left) and K18 (right) added to TBE vesicles at molar protein:phospholipid ratios of 1:20 (top) and 1:160 (bottom) shows that protein/phospholipid complexes can form in the presence of TBE. The scale bars are 100 nm. (**b**) Native PAGE analysis of protein/phospholipid complexes formed by Tau (left) and K18 (right) after 24 h of incubation with TBE vesicles at a molar protein:phospholipid ratio of 1:20. The monomer bands are indicated by asterisks, and the presence of higher molecular weight bands, indicated by arrows, demonstrates that the complexes contain oligomerized proteins. (**c**) CD spectra of Tau (upper panel) and K18 (lower panel) alone (blue) and in the presence of TBE vesicles (red) at 0 h (dashed lines) and 24 h (solid lines) at a molar protein:phospholipid ratio of 1:160. The formation of protein/phospholipid complexes is accompanied by an increase in secondary structure content. The signal is shown from 210 nm (Tau) and 208 nm (K18) due to signal saturation below these wavelengths in the presence of high ratios of TBE vesicles. (**d**) Co-sedimentation assay of TBE vesicles mixed with K18 (top) and Tau (bottom) at a molar protein:phospholipid ratio of 1:160. When incubated alone (left), all proteins remain in the supernatant (sup.), but in the presence of TBE vesicles (right), the proteins partially co-sediment with the vesicles to the pellet fraction (pel.), indicative of membrane binding.

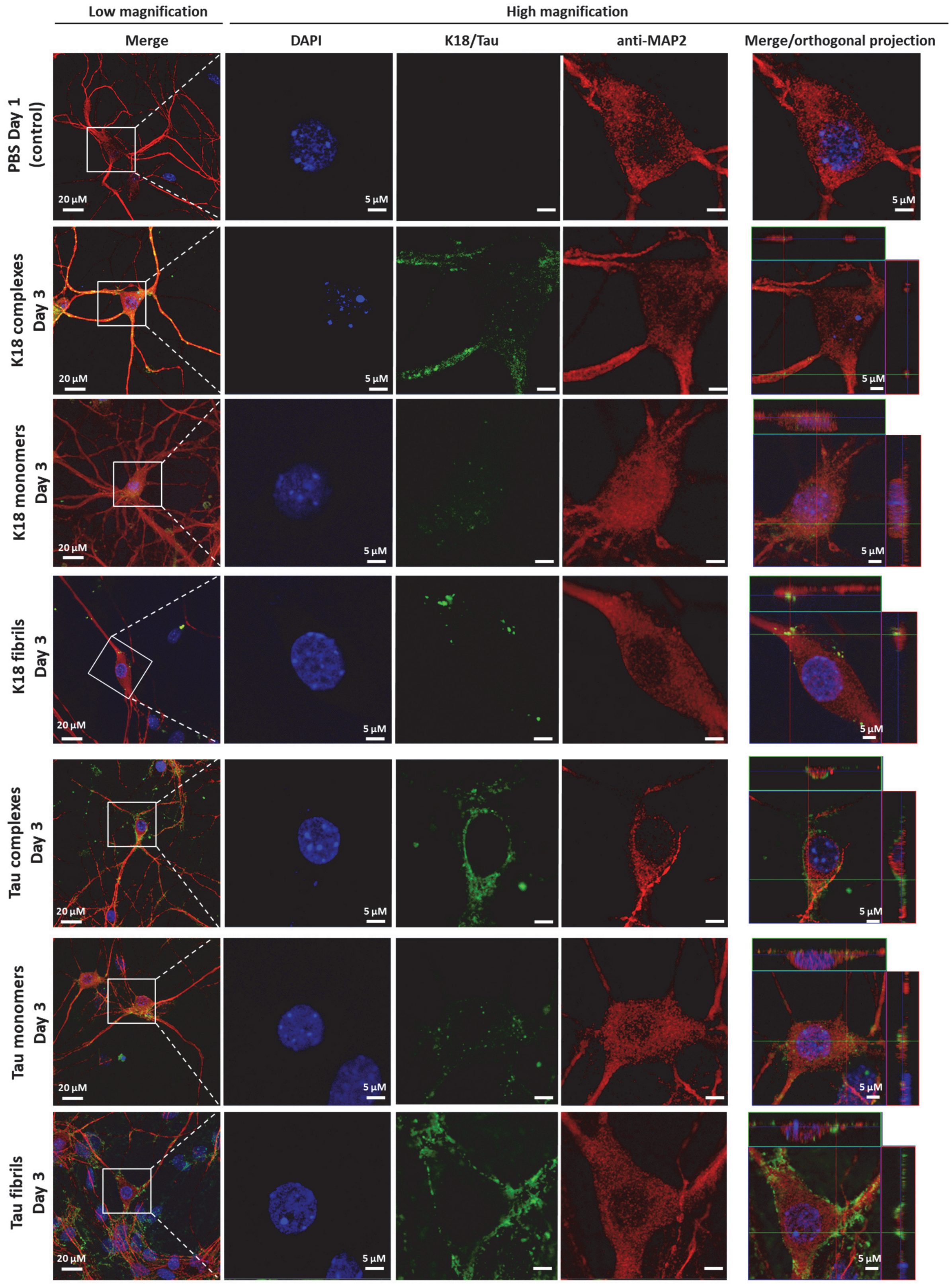

Supplementary Figure 4│Internalization of Tau and K18 monomers, fibrils and protein/phospholipid complexes at 3 days analyzed by immunochytochemistry.

Hippocampal primary neurons were treated with PBS, 1 µM Tau or Oregon Green-labeled K18 (K18-OG) monomers, fibrils or protein/phospholipid complexes for 3 days and processed for immunocytochemistry. From left to right: overview image, DAPI staining to show the nucleus (blue), K18-OG or staining with anti-Tau antibody Tau13 (green), staining with anti-MAP2 antibody identifying neurons (red), and orthogonal projections. In neurons treated with protein/phospholipid complexes

containing K18-OG, K18 localizes to the plasma membrane and is internalized by primary neurons after 3 days, as evidenced by the co-staining with MAP2. In neurons treated with monomeric K18-OG, K18 is internalized and is diffuse in the cytoplasmic compartment and in small punctae after 3 days. In neurons treated with K18-OG fibrils, K18 localizes to the membrane in the form of large punctae that are internalized by primary neurons. In neurons treated with Tau protein/phospholipid complexes, after 3 days, Tau is no longer observed inside the neurons, but strongly localizes to the plasma membrane. In neurons treated with monomeric Tau, Tau is internalized and is diffuse in the cytoplasmic compartment and in small punctae. In neurons treated with sonicated Tau fibrils, Tau localizes to the plasma membrane and is not internalized at 3 days post-treatment. The scale bars are 20 µm (low magnification) and 5 µm (high magnification).

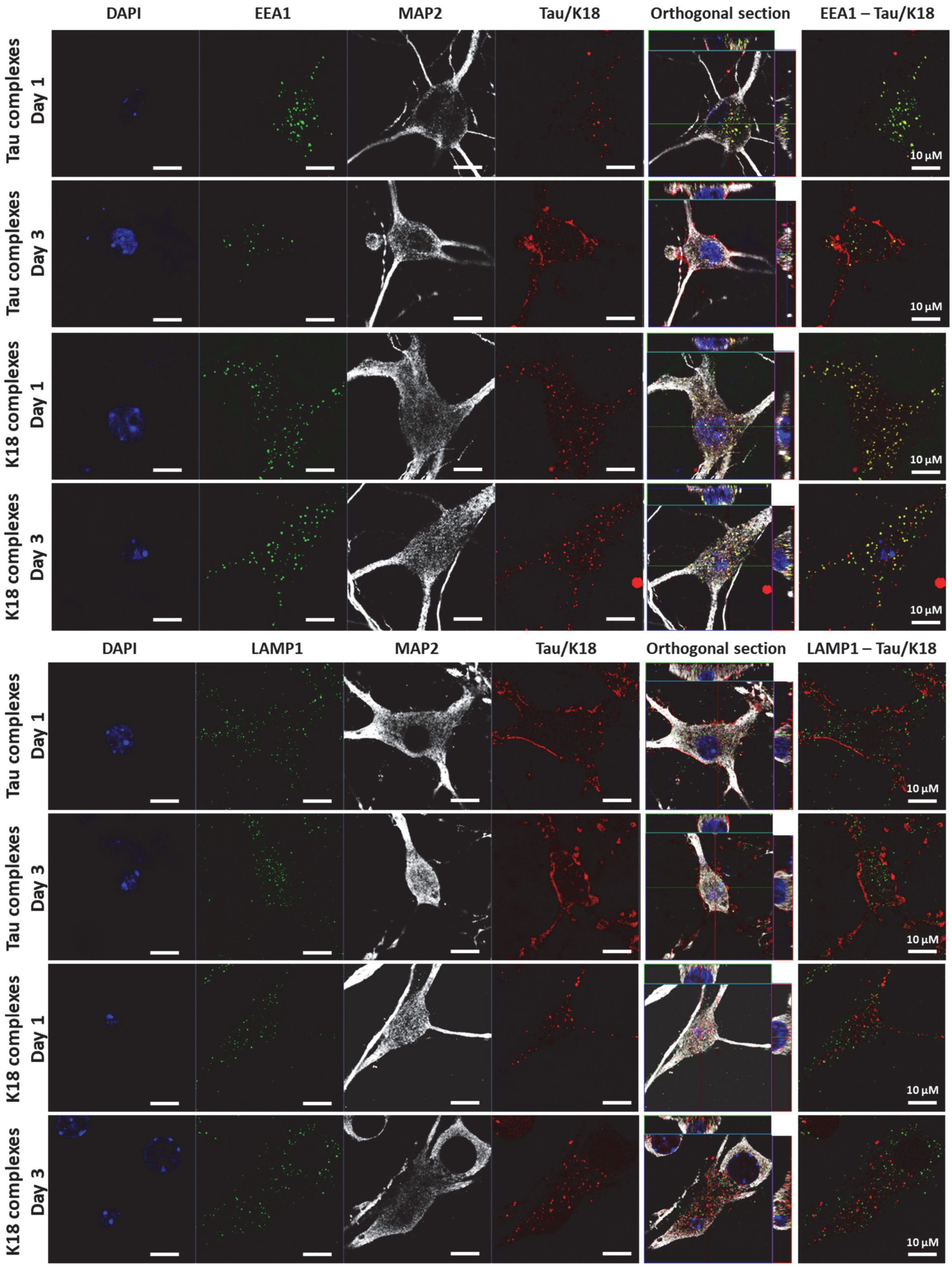

Supplementary Figure 5 | Colocalization of K18 protein/phospholipid complexes with endolysosomal markers. Hippocampal primary neurons were treated with 1 µM TAMRA-labeled Tau or K18 protein/phospholipid complexes for 1 or 3 days and processed for immunocytochemistry. Top panels, from left to right: DAPI staining to show the nucleus (blue), staining with anti-EEA1 antibody to identify early endosomes (green), staining with anti-MAP2 antibody to identify neurons (white), TAMRA-labeled Tau or K18 (red) and orthogonal projections. Bottom panels: from left to right: DAPI staining to show

the nucleus (blue), staining with anti-LAMP1 antibody to identify late endosomes and lysosomes (green), staining with anti-MAP2 antibody to identify neurons (white), TAMRA-labeled Tau or K18 (red) and orthogonal projections. Immunocytochemical analysis of neurons treated with Tau or K18 protein/phospholipid complexes containing TAMRA-labeled Tau or K18 at 1 and 3 days show that the complexes are internalized and found in small punctae that often co-localize with the EEA1 staining, as observed by the merged yellow color in the orthogonal projection. Tau and K18 protein/phospholipid complexes do not co-localize with LAMP1. The scale bars are 10 µm.

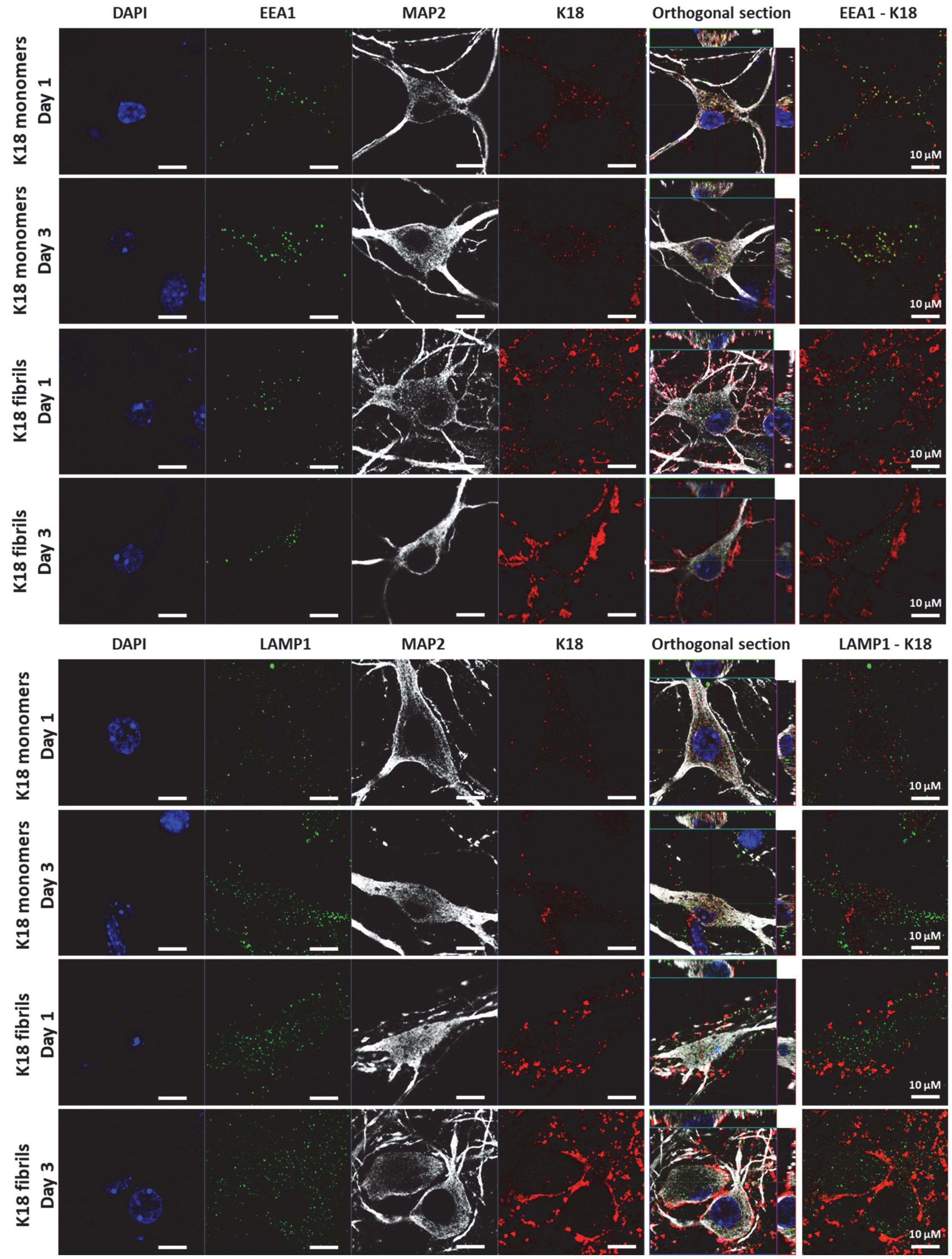

Supplementary Figure 6│Colocalization of K18 monomers and fibrils with endolysosomal markers.
Hippocampal primary neurons were treated with 1 µM TAMRA-labeled K18 species for 1 or 3 days and processed for immunocytochemistry. Top panels, from left to right: DAPI staining to show the nucleus (blue), staining with anti-EEA1 antibody to identify early endosomes (green), staining with anti-MAP2 antibody to identify neurons (white), TAMRA-labeled K18 (red) and orthogonal projections. Bottom panels: from left to right: DAPI staining to show the nucleus (blue), staining with anti-LAMP1 antibody to identify late endosomes and lysosomes (green), staining with anti-MAP2 antibody to identify neurons (white), TAMRA-labeled K18 (red) and orthogonal projections. Immunocytochemical analysis of neurons treated with K18 monomer and fibrils containing TAMRA-labeled K18 at 1 and 3 days show that monomeric K18 is internalized and found in small punctae that partially co-localize with EEA1. Fibrillar K18 localizes to the plasma membrane and does not co-localize with EEA1. Neither of these two K18 species co-localizes with LAMP1. The scale bars are 10 µm.

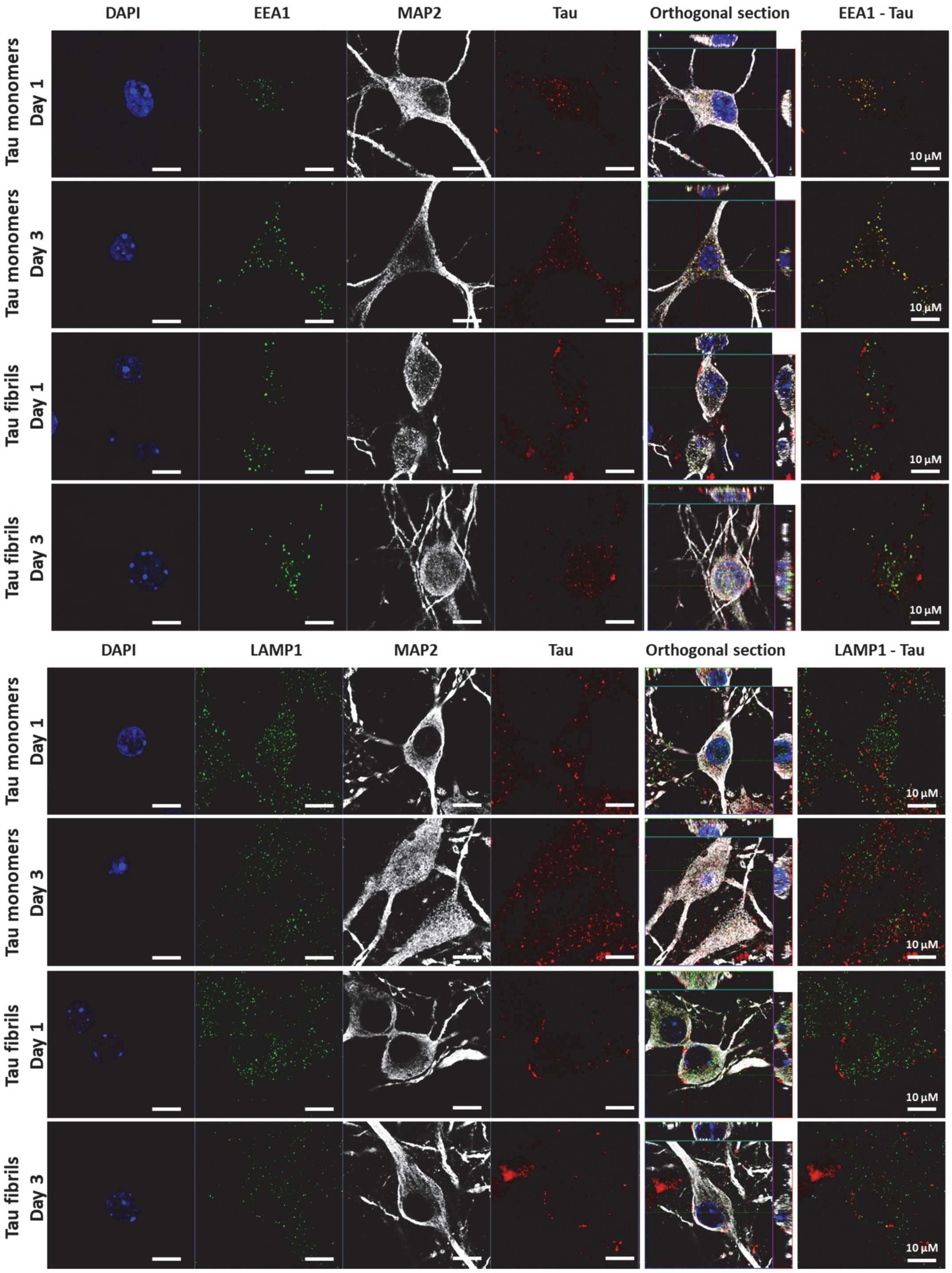

Supplementary Figure 7 | Colocalization of Tau monomers and fibrils with endolysosomal markers. Hippocampal primary neurons were treated with 1 µM TAMRA-labeled Tau for 1 or 3 days and processed for immunocytochemistry. Top panels, from left to right: DAPI staining to show the nucleus (blue), staining with anti-EEA1 antibody to identify early endosomes (green), staining with anti-MAP2 antibody to identify neurons (white), TAMRA-labeled Tau (red) and orthogonal projections. Bottom panels: from left to right: DAPI staining to show the nucleus (blue), staining with anti-LAMP1 antibody to identify late endosomes and lysosomes (green), staining with anti-MAP2 antibody identifying neurons (white), TAMRA-labeled Tau (red) and orthogonal projections. Immunocytochemical analysis of neurons treated with Tau monomer and fibrils containing TAMRA-labeled Tau at 1 and 3 days shows that monomeric Tau is internalized and found in small punctae that partially co-localize with EEA1. Fibrillar Tau localizes mainly to the plasma membrane and does not co-localize with EEA1. Neither of these two Tau species co-localizes with LAMP1. The scale bars are 10 µm.

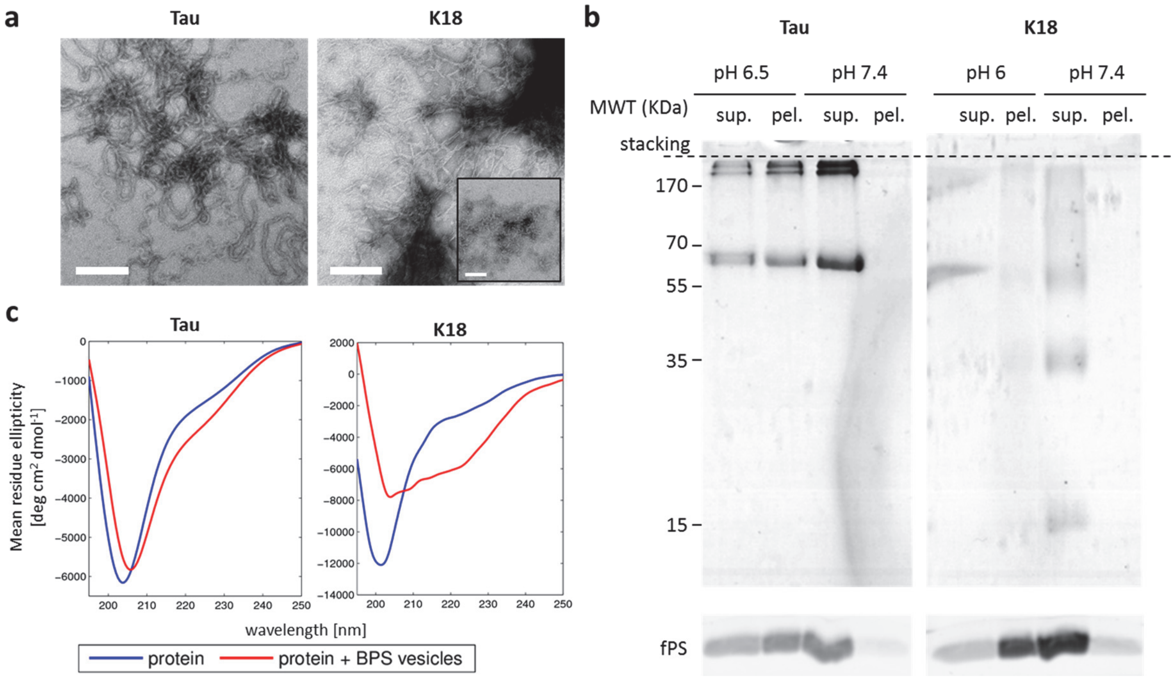

Supplementary Figure 8 | Protein/phospholipid complexes can convert into extended aggregates at pH 6.5.

(**a**) Negative-stain EM images of Tau (left) and K18 (right) protein/phospholipid complexes taken 24 h after changing the pH to 6.5 or 6, respectively. While Tau forms elongated filamentous structures exclusively, K18 forms both elongated and smaller globular structures (inset). The scale bars are 200 nm. (**b**) Corresponding sedimentation assays. SDS-PAGE analysis shows that for both Tau and K18 samples, only the protein/phospholipid complexes that were incubated at pH 6.5 are found in the pellet, indicating that larger species were formed. The presence of phospholipids in the pellet was established by using NBD-labeled phosphatidylserine (bottom), denoted fPS, and imaging of the gel with a Typhoon trio fluorescence scanner. (**c**) CD of Tau (left) or K18 (right) incubated alone (blue line) or with BPS vesicles (red line) first at pH 7.4 for 24 h and then at pH 6.5 (Tau) or 6 (K18) for another 24 h.

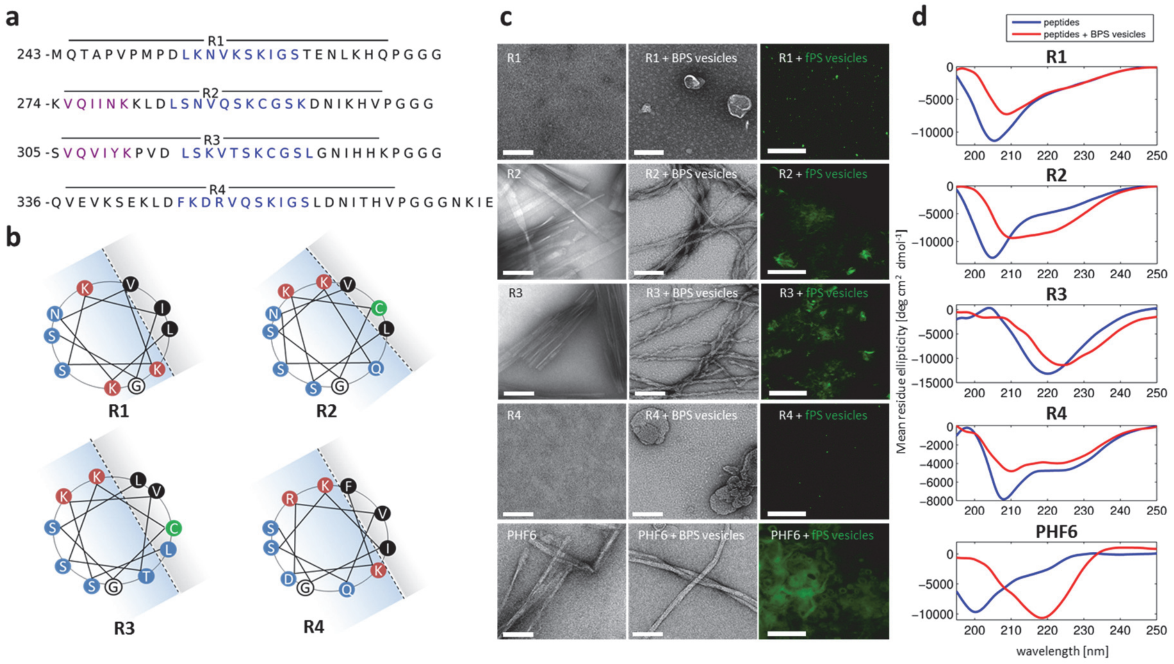

Supplementary Figure 9 | Interaction of the microtubule-binding repeat-derived peptides with negatively charged vesicles. (**a**) Amino acid sequence of K18. The lines indicate the peptides used in this study, which encompass microtubule-binding repeats R1 to R4. The sequences for PHF6 and PHF6* are shown in purple and those for the amphipathic α-helices in blue. (**b**) Helical wheel representation of the microtubule-binding repeat sequences, showing that they can potentially form amphipathic helices. Hydrophilic residues are shown in black, hydrophobic residues in blue, positively charged residues in red, cysteine in green, and glycine (which has no side chain) in white. The hydrophobic and hydrophilic faces of the helices are depicted in light grey and blue, respectively. (**c**) Negative-stain EM and fluorescence microscopy images of microtubule-binding repeats R1, R2, R3, R4 and PHF6 peptides incubated for 3 days either alone or with BPS vesicles at a molar ratio of 1:1. For the EM images, the scale bars are 100 nm. For fluorescence microscopy, the five peptides were incubated with vesicles containing 1% fluorescent NBD-phospholipids. The scale bars are 20 µm. Upon incubation with BPS vesicles, microtubule-binding repeat peptides R2 and R3 formed large and heterogeneous fibrils. The differences in morphology between fibrils formed in the presence and absence of phospholipids suggest that interaction with the vesicles altered the way the peptides aggregated or that the phospholipids became incorporated into the fibrils. To test the latter hypothesis, the peptides were co-incubated with vesicles containing NBD-labeled fluorescent phospholipids. Under these conditions, we observed the formation of fluorescent aggregates, presumably representing fibrils, only in samples containing vesicles and R2 or R3, consistent with the incorporation of phospholipids into the fibrils. Lipid incorporation could be due to the aggregation-prone PHF6* and PHF6 peptides being located adjacent to the amphipathic helix-forming sequences of R2 and R3. (**d**) CD spectra of the five peptides alone (blue) and after incubation with BPS vesicles for 24 h (red).

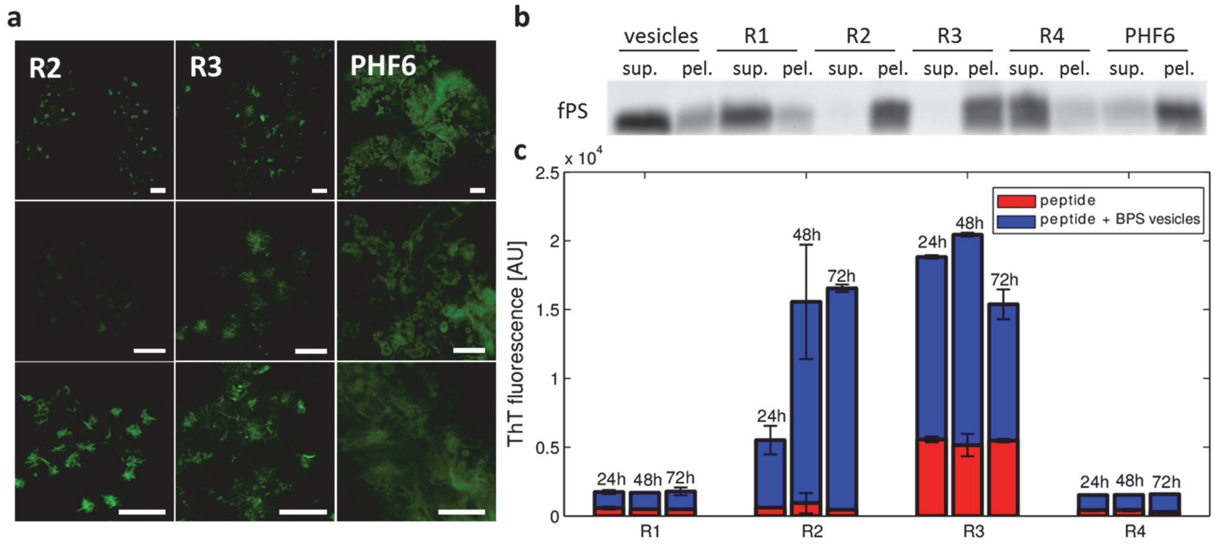

Supplementary Figure 10 | *In vitro* aggregation of the microtubule-binding repeat peptides R2 and R3 and the PHF6 peptide in the presence of BPS vesicles.

(**a**) Fluorescence microscopy images of the R2, R3 and PHF6 aggregates formed when incubated for 24 h with BPS vesicles containing 1% NBD-labeled fluorescent phospholipids. The scale bars are 20 µm. (**b**) SDS-PAGE analysis of the co-sedimentation assays. The R1, R2, R3, R4 and PHF6 peptides were incubated for 24 h with BPS vesicles containing 1% NBD-labeled fluorescent phospholipids (fPS) at a molar peptide:phospholipid ratio of 1:1. The samples were then centrifuged for 5 min at 5000 g and the pellets and supernatants were run on SDS-PAGE gels that were imaged with a the Typhoon trio fluorescent scanner (GE Healthcare). The presence of fluorescent lipids in the pellet fractions (pel.) indicates that the R2, R3 and PHF6 peptides formed large structures that contain phospholipids. (**c**) Thioflavin T fluorescence of peptides R1, R2, R3 and R4 incubated either alone (red) or with BPS vesicles at a molar ratio of 1:1 (blue) for the indicated time periods shows that upon binding to phospholipids, peptides R2 and R3, but not R1 and R4, form stacked β-sheet structures that strongly bind ThT. The presence of the PHF6* and PHF6 hexapeptides in R2 and R3 might explain the conversion of the vesicles into mixed peptide/phospholipid fibrils in the presence of R2 and R3, but not R1 and R4.

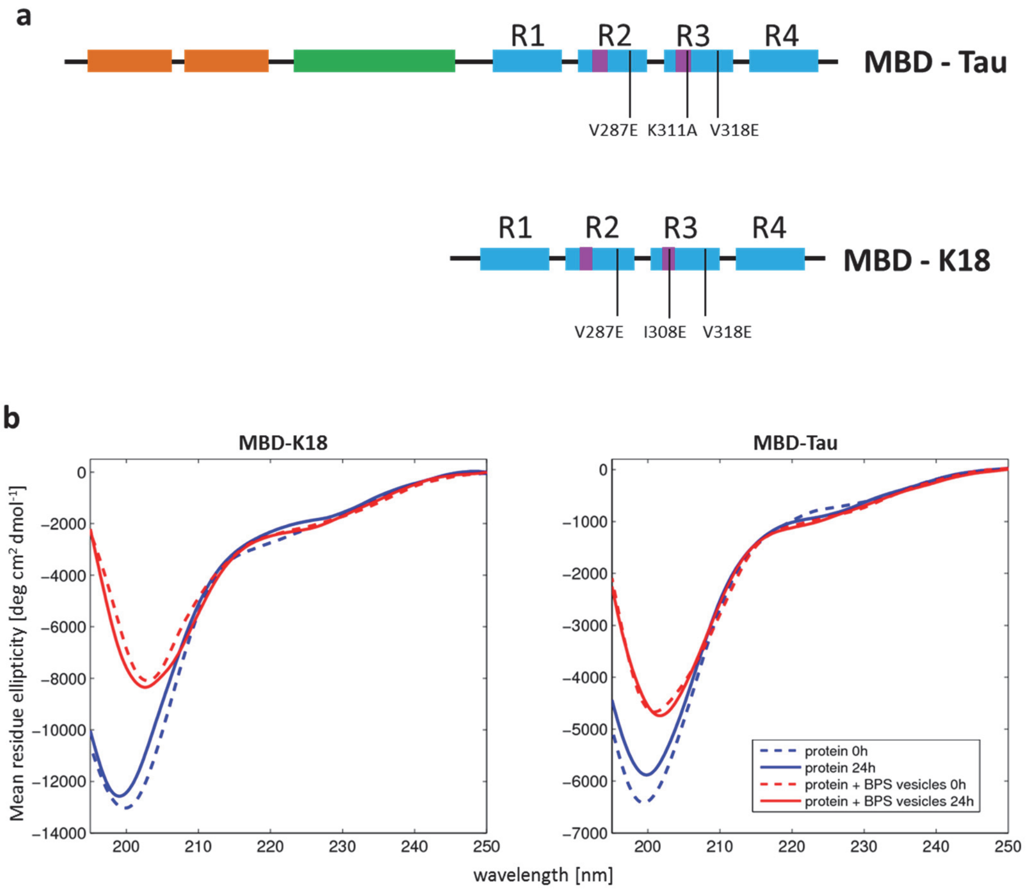

Supplementary Figure 11│Interaction sequences and structural characterization of the Tau and K18 membrane binding-deficient (MBD) mutants.

(**a**) Membrane binding-deficient (MBD) Tau contains the three mutations V287E, V318E and K311A. In MBD-K18, the K311A mutation is replaced by the I308E mutation. (**b**) CD spectra of MBD-K18 (V287E/I308E/V318E) (left) and MBD-Tau (287E/K311A/V318E) (right) incubated for 24 h alone (blue) and in the presence of BPS vesicles (red) at 0 h (dashed lines) and 24 h (solid lines) at a molar protein:phospholipid ratio of 1:20 show that the presence of vesicles does not induce substantial changes in secondary structure, consistent with the inability of these mutants to form protein/phospholipid complexes.

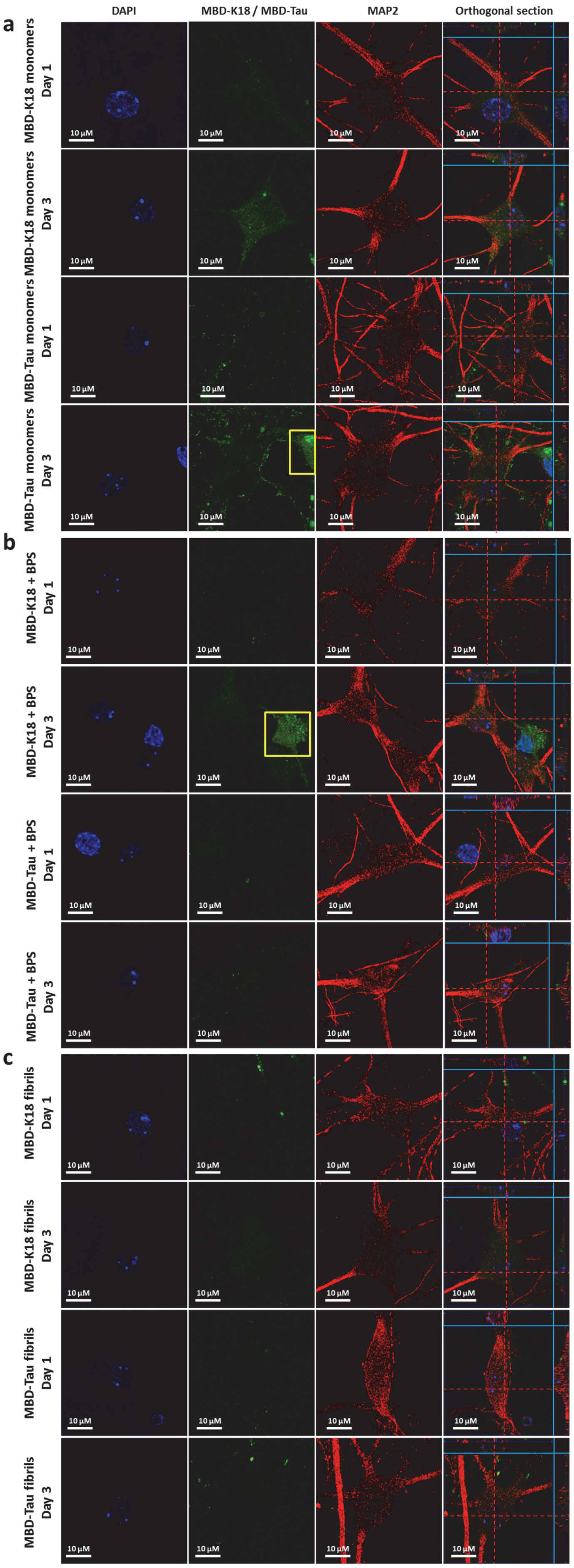
a
b
c
DAPI
MBD-K18 / MBD-Tau
MAP2
Orthogonal section
MBD-K18 monomers Day 1
MBD-K18 monomers Day 3
MBD-Tau monomers Day 1
MBD-Tau monomers Day 3
MBD-K18 + BPS Day 1
MBD-K18 + BPS Day 3
MBD-Tau + BPS Day 1
MBD-Tau + BPS Day 3
MBD-K18 fibrils Day 1
MBD-K18 fibrils Day 3
MBD-Tau fibrils Day 1
MBD-Tau fibrils Day 3
10 µM

Supplementary Figure 12│Internalization of MBD-Tau and MBD-K18 monomers, fibrils and mixed with BPS vesicles analyzed by immunocytochemistry.

(**a**) Internalization of monomeric MBD-Tau and MBD-K18. Hippocampal primary neurons were treated with 1 µM monomeric TAMRA-labeled MBD-Tau or MBD-K18 for 1 or 3 days and processed for immunocytochemistry. From left to right: DAPI staining of the nucleus (blue), TAMRA-labeled MBD-Tau or MBD-K18 (green), anti-MAP2 antibody staining to identify neurons (red), and merged orthogonal projections. Images of neurons treated with monomeric MBD-Tau or MBD-K18 show low level of diffuse and punctate (and membrane-bound for the full-length protein) staining at three days. It is noteworthy that all species are significantly taken up by non-neuronal cells (yellow box). (**b**) Internalization of MBD-Tau and MBD-K18 that were incubated with BPS vesicles. Hippocampal primary neurons were treated for 1 or 3 days with 1 µM TAMRA-labeled MBD-Tau or MBD-K18 that were incubated with BPS vesicles and processed for immunocytochemistry. From left to right: DAPI staining of the nucleus (blue), TAMRA-labeled MBD-Tau or MBD-K18 (green), anti-MAP2 antibody staining to identify neurons (red), and merged orthogonal projections. Images of neurons treated with MBD-Tau or MBD-K18 that were incubated with BPS vesicles show a very low level of diffuse and punctate staining at three days**.** (**c**) Internalization of MBD-Tau and MBD-K18 fibrils. Hippocampal primary neurons were treated with 1 µM fibrillar TAMRA-labeled MBD-Tau or MBD-K18 for 1 or 3 days and processed for immunocytochemistry. From left to right: DAPI staining of the nucleus (blue), TAMRA-labeled MBD-Tau or MBD-K18 (green), anti-MAP2 antibody staining to identify neurons (red), and merged orthogonal projections. Images of neurons treated with fibrillar MBD-Tau or MBD-K18 show a low level of membrane-proximal large punctae, mainly after 3 days of treatment. It is noteworthy that all species are significantly taken up by non-neuronal cells (yellow box). The scale bars are 200 nm.

Supplementary Figure 13 | Uncropped images of gels with the marker lanes

Figure 1b:

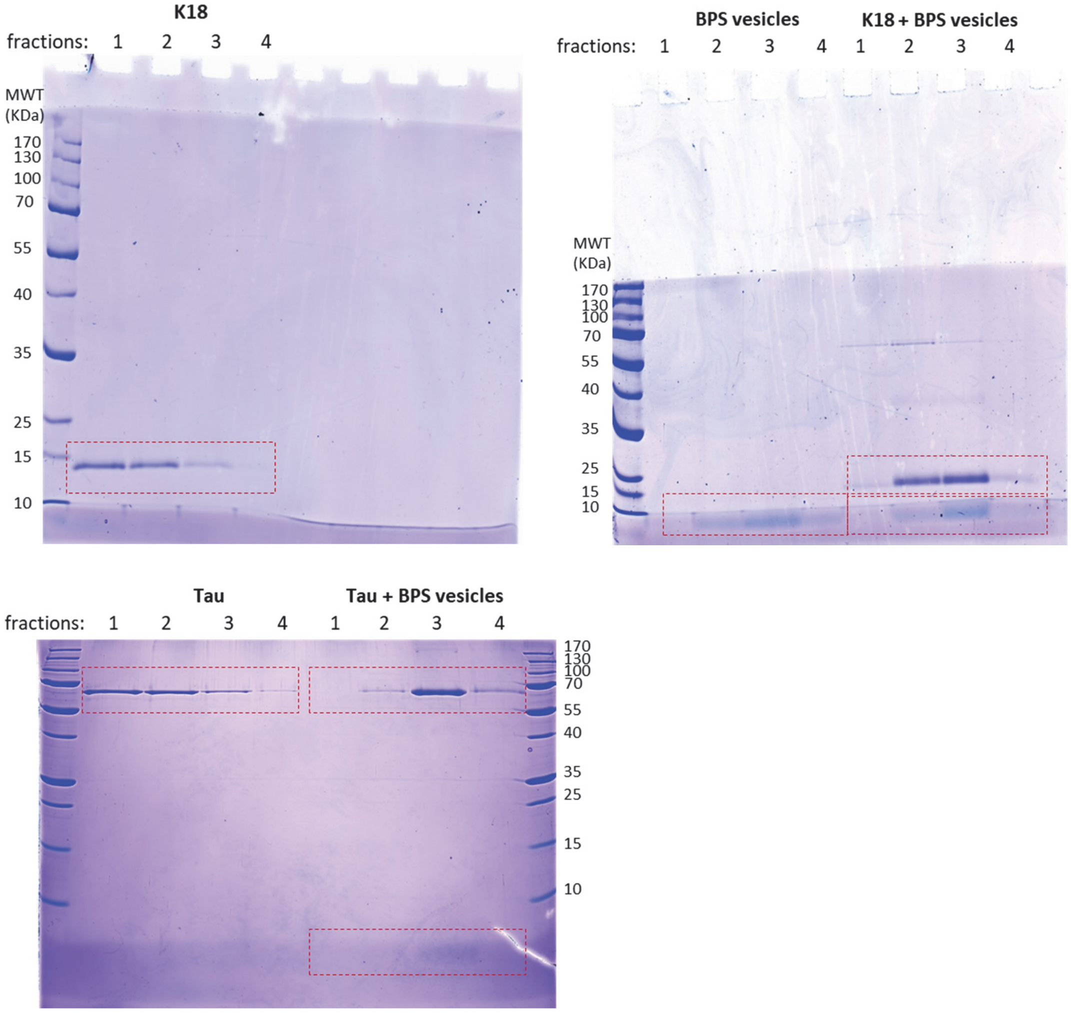

Figure 2c:

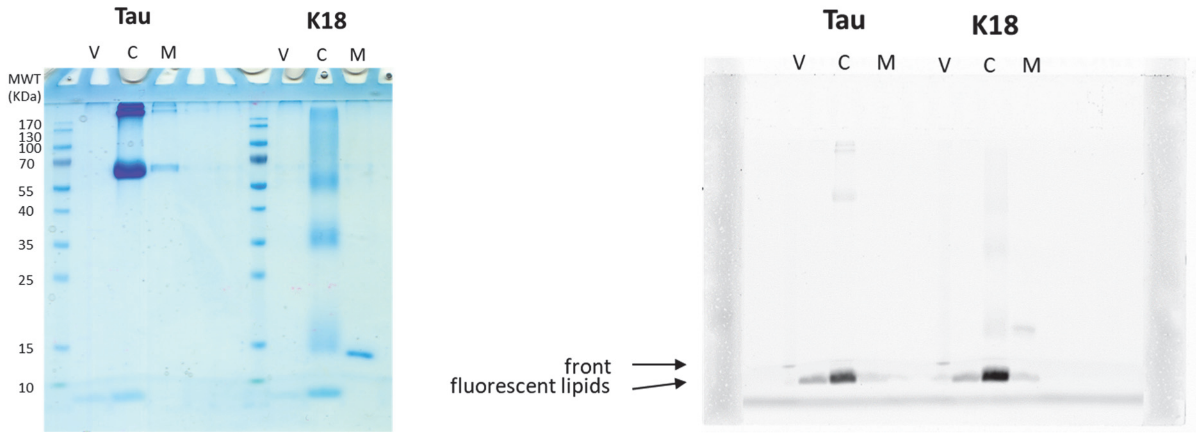

Figure 3a:

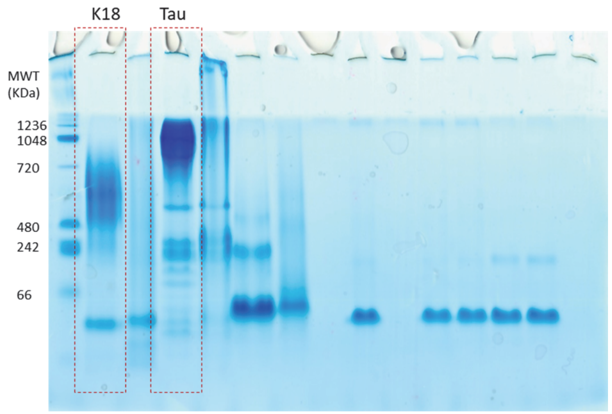

Figure 4b:

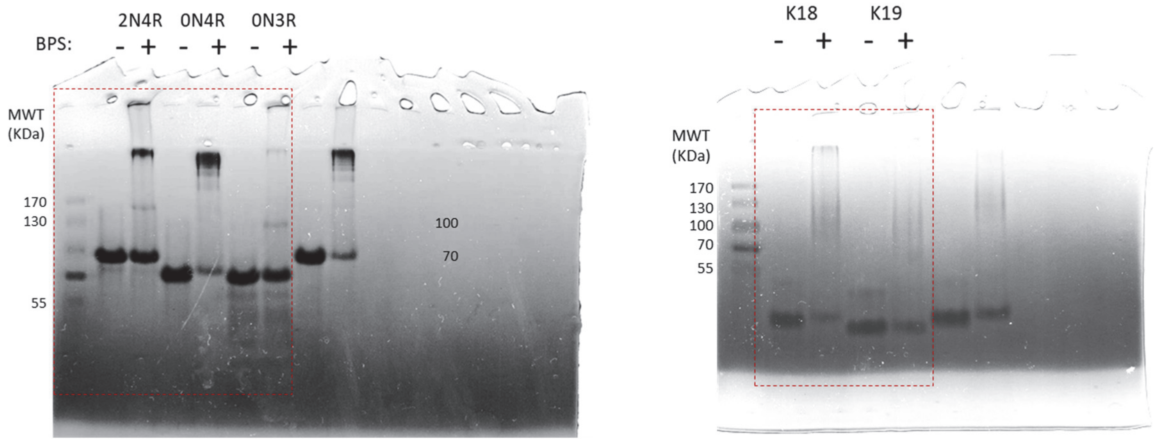

Figure 4c:

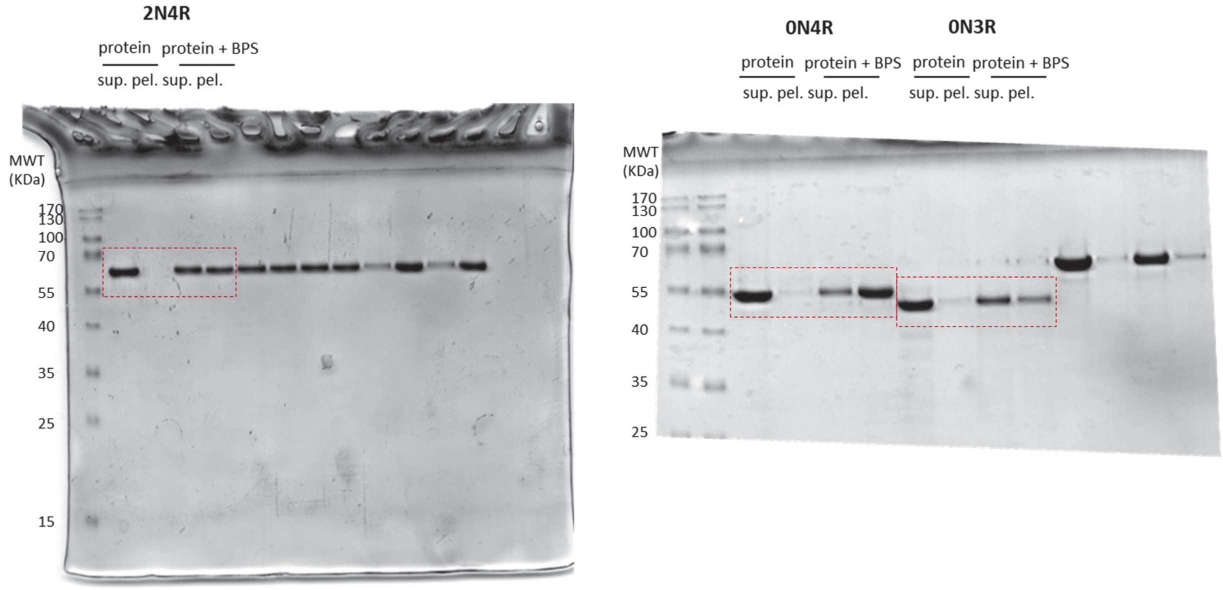

Figure 7b:

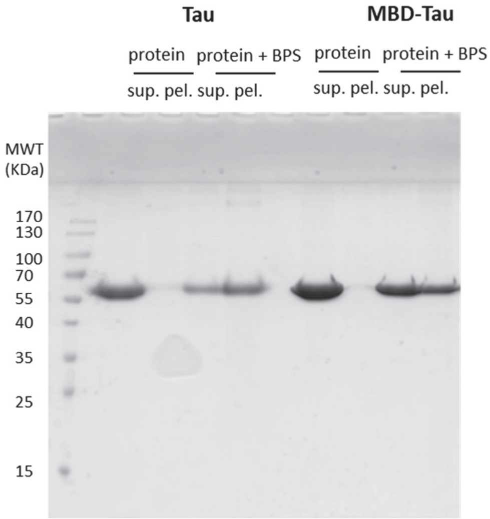

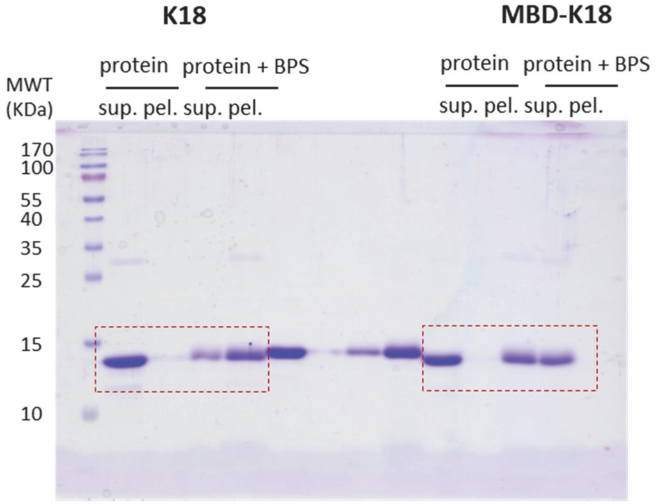

Supplementary Figure 3b:

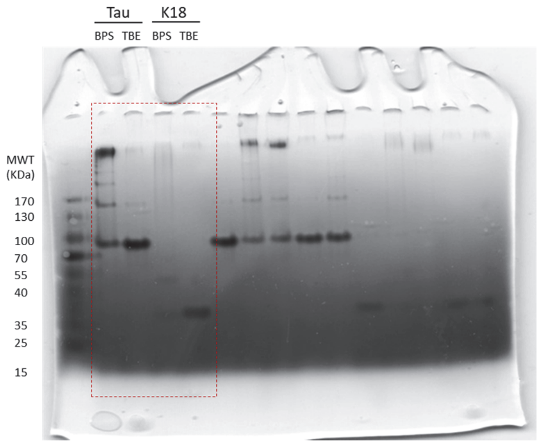

Supplementary Figure 3d:

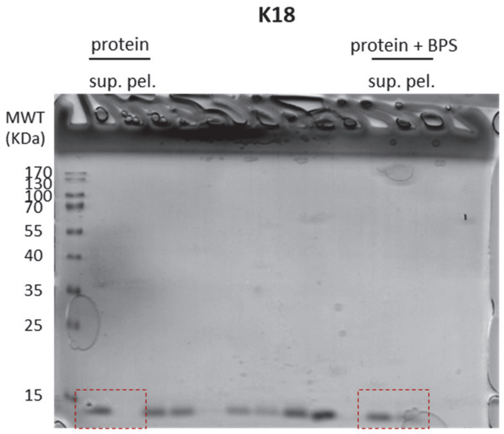

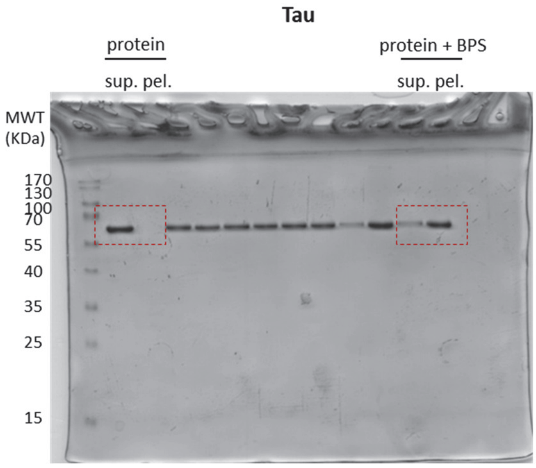

Supplementary Figure 8b

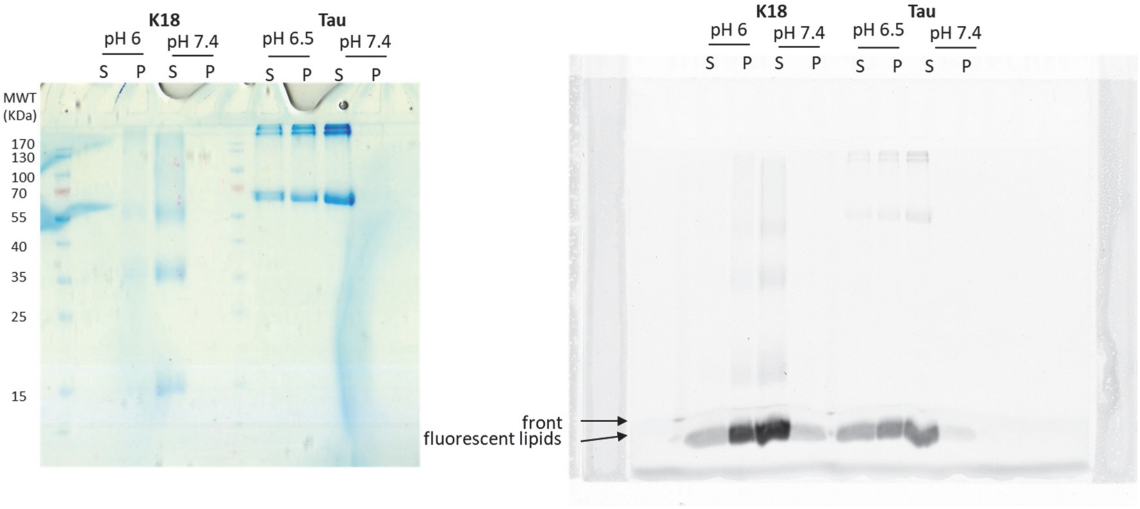

Supplementary Figure 10b

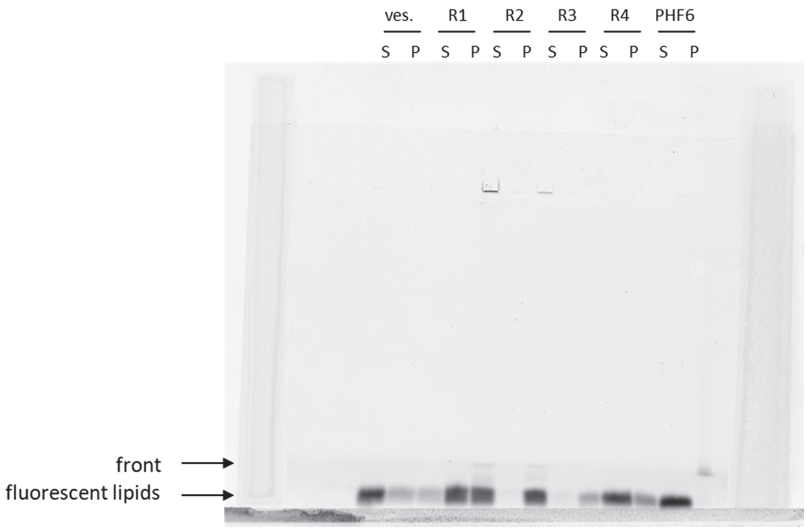

Supplement: Supplementary file 1 — Supplementary Information [file 41467_2017_1575_MOESM1_ESM.pdf]
